# Mehrere Triplett‐Metall‐zentrierte Jahn–Teller‐Isomere bestimmen die temperaturabhängigen Lumineszenzlebensdauern in [Ru(bpy)_3_]^2+^

**DOI:** 10.1002/ange.202308803

**Published:** 2023-09-15

**Authors:** David Hernández‐Castillo, Roland E. P. Nau, Marie‐Ann Schmid, Stefanie Tschierlei, Sven Rau, Leticia González

**Affiliations:** ^1^ Institute of Theoretical Chemistry Faculty of Chemistry University of Vienna Währinger Str. 17 1090 Vienna Austria; ^2^ Doctoral School in Chemistry (DoSChem) University of Vienna Währinger Straße 42 1090 Vienna Austria; ^3^ Institute of Inorganic Chemistry I Ulm University Albert-Einstein-Allee 11 89081 Ulm Germany; ^4^ Technische Universität Braunschweig Department of Energy Conversion, Institute of Physical and Theoretical Chemistry Rebenring 31 38106 Braunschweig Germany; ^5^ Vienna Research Platform Accelerating Photoreaction Discovery University of Vienna Währinger Straße 17 1090 Vienna Austria

**Keywords:** Koordinationschemie, Ruthenium-Verbindungen, Photophysik, Temperaturanhängige Photolumineszenz, Lumineszenzlebensdauer

## Abstract

Ein genaues Verständnis der Faktoren, welche die Lumineszenzlebensdauer von Übergangsmetallverbindungen bestimmen, ist für Anwendungen in der Photokatalyse und der photodynamischen Therapie von entscheidender Bedeutung. Die im Falle von [Ru(bpy)_3_]^2+^ (bpy=2,2’‐Bipyridin) allgemein akzeptierte Theorie besagt, dass die Emissionslebensdauer durch Optimierung der Energiebarriere zwischen dem emittierenden Triplett‐Zustand des Metall‐Liganden‐Ladungstransfers (^3^MLCT) und dem thermisch aktivierten Triplett‐Zustand des Metall‐Zentrums (^3^MC), oder der Energielücke zwischen beiden Zuständen gesteuert werden kann. Hier zeigen wir, dass dies nicht allgemeingültig ist. Darüber hinaus demonstrieren wir, dass die Betrachtung eines einzelnen Relaxationspfades, der vom energetisch niedrigsten Minimum aus bestimmt wird, zu falschen Vorhersagen der temperaturabhängigen Emissionslebensdauer führt. Stattdessen erhalten wir eine ausgezeichnete Übereinstimmung mit den experimentellen temperaturabhängigen Lebensdauern, wenn ein erweitertes kinetisches Modell herangezogen wird, welches alle Pfade im Zusammenhang mit mehreren Jahn–Teller‐Isomeren und ihren effektiven Reaktionsbarrieren beinhaltet. Diese Konzepte sind für das Design weiterer lumineszierender Übergangsmetallkomplexe mit individuell angepassten Emissionslebensdauern auf der Grundlage theoretischer Vorhersagen unerlässlich.

## Graphical abstract

Die zeitabhängigen Emissionslebensdauern von [Ru(bpy)_3_]^2+^ stimmen nur dann mit den experimentellen Werten überein, wenn mehrere Pfade mit Jahn–Teller‐Isomeren der angeregten ^3^MC Zustände berücksichtigt werden.

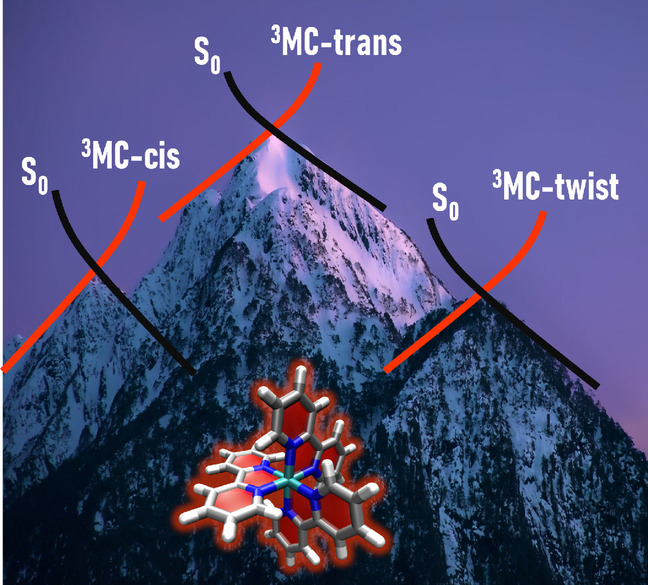

## Einleitung

Ruthenium‐(Ru)‐Polypyridin‐Komplexe mit langer Lumineszenzlebensdauer sind in vielen Forschungsbereichen von großer Bedeutung: von der Entwicklung erneuerbarer Energien, einschließlich der $H_{2}$ ‐Produktion,[^1^, ^2^] CO_2_‐Reduktion,[^3^, ^4^] und farbstoffsensibilisierten Solarzellen,^[5]^ bis hin zur synthetischen Organophotoredoxkatalyse^[6]^ und photodynamischen Therapie.^[7]^ Die Lebensdauer des lumineszierenden Zustands wird durch konkurrierende Deaktivierungsreaktionen aus dem niedrigsten Triplett‐Elektronenzustand bestimmt. Dieser weist bei solchen Komplexen den Charakter eines Metall‐Liganden‐Ladungstransfers (^3^MLCT) auf, d. h. eines Zustands, bei dem ein d‐Elektron des Ru‐Zentrums auf ein Orbital des Bipyridyl‐Liganden übertragen wurde.

**Figure 1:**
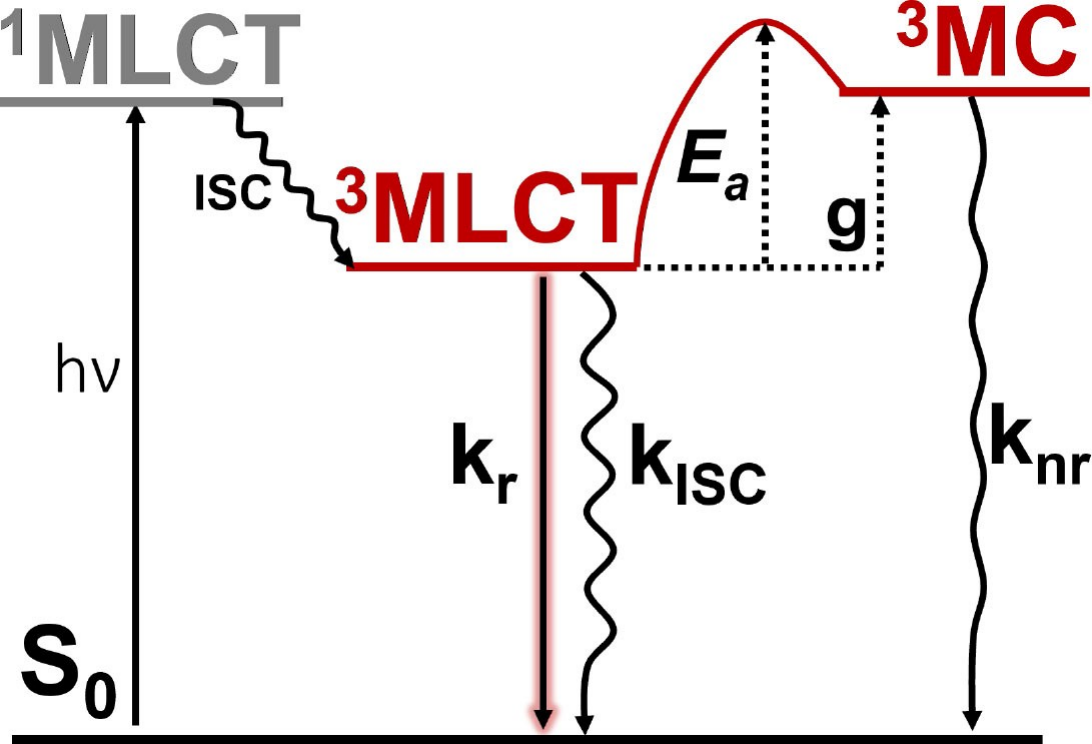
Vereinfachtes Jablonski‐Diagramm für Ru‐Polypyridyl‐Komplexe. Bei Bestrahlung werden Singulett‐Metall‐zu‐Ligand‐Ladungstransfer‐Zustände (^1^MLCT) angeregt, die durch Intersystem‐Crossing (ISC) in die strahlenden ($k_{r}$ ) ^3^MLCT‐Zustände relaxieren. Wenn genügend thermische Energie zur Verfügung steht, um die Barriere *E*_a_ zu überwinden, kann eine Relaxation vom niedrigsten ^3^MLCT‐Zustand zu Triplett‐Metall‐zentrierten (^3^MC) Zuständen stattfinden, von wo aus das System strahlungsfrei ($k_{\mathrm{nr}}$ ) in den elektronischen Grundzustand zurückfällt. *g* ist definiert als die Energielücke zwischen den niedrigsten ^3^MLCT‐ und ^3^MC‐Zuständen.

In einigen Beispielen wurde die Triplett‐Lebensdauer von Ru‐basierten Verbindungen auf Mikrosekunden ausgedehnt.^[8]^ Trotz aussichtsreicher experimenteller Ergebnisse von ausgeweiteten π‐Orbitalen von Bipyridin, die zu einer Verlängerung der Lebensdauer lumineszierender Zustände führen, bleibt es eine Hürde, die Ergebnisse aus der Theorie umzusetzen, um Liganden mit passenden Eigenschaften zu entwickeln. Die Herausforderung liegt darin Theorie und Experiment zu verbinden, was die Entwicklung fortschrittlicher photodynamischer Therapiemittel für hypoxische Umgebungen^[9]^ oder Anwendungen, die kleine Mengen von Katalysatoren mit langen Lebensdauern im angeregten Zustand erfordern, verhindert. Eine zusätzliches Problem besteht darin, dass bei Raumtemperatur, der idealen Arbeitsbedingung für die meisten Anwendungen, die Lebensdauer und die Emissionsquantenausbeute der ^3^MLCT‐Zustände exponentiell abnimmt.[^10^, ^11^, ^12^] Dies liegt daran, dass mit zunehmender Temperatur die Relaxation vom niedrigsten ^3^MLCT‐Zustand zu thermisch aktivierten und nicht emittierenden Metall‐zentrierten Triplett‐Zuständen (^3^MC) mit der Emission konkurriert (siehe Abbildung 1). Letztendlich führt dies zu einer strahlungslosen Relaxation in den Grundzustand. Jüngste Ergebnisse haben gezeigt, dass eine Erhöhung der Temperatur auf bis zu 50 °C bei der lichtgetriebenen Zwei‐Elektronen‐Reduktionskatalyse zu einer 6,5‐fachen Beschleunigung der katalytischen Leistung führt,^[13]^ und damit den Weg für die Herstellung von Photokatalysatoren, welche thermischen Katalysatoren überlegen sind, ebnet. Daher ist es von größter Bedeutung, die primären photochemischen Reaktionsschritte und die Faktoren zu verstehen, welche die konkurrierenden Deaktivierungspfade bestimmen, um die Emissionslebensdauer und letztlich die Leistung von Photokatalysatoren zu optimieren.

Der prototypische Tris(bipyridin)‐Ru‐Komplex, ${\left[\mathrm{Ru}{\left(\mathrm{bpy}\right)}_{3}\right]}^{2+}$ (bpy=2,2’‐Bipyridin), ist das bei weitem meist verwendete Molekül im Zusammenhang mit temperaturabhängigen Phosphoreszenzlebensdauerphänomenen. Erkenntnisse aus der Untersuchung von ${\left[\mathrm{Ru}{\left(\mathrm{bpy}\right)}_{3}\right]}^{2+}$ werden häufig direkt auf verwandte Ru‐Derivate^[14]^ und andere lumineszierende Übergangsmetallkomplexe übertragen,[^15^, ^16^] auch wenn es keine Anhaltspunkte dafür gibt, dass das Verhalten von ${\left[\mathrm{Ru}{\left(\mathrm{bpy}\right)}_{3}\right]}^{2+}$ verallgemeinert werden kann. Ein experimenteller Einblick in die Deaktivierung vom niedrigsten ^3^MLCT‐Zustand wird in der Regel durch Photolumineszenzmessungen bei variierenden Temperaturen gewonnen. Die erhaltenen Emissionslebensdauern werden dann mit Hilfe von Gleichung 1 gegen die Temperatur aufgetragen.

(1) $$\tau_{\mathrm{emission}}=\frac{1}{k_{r}+k_{\mathrm{ISC}}+k_{\mathrm{nr}}}$$

In dieser Gleichung (siehe Abbildung 1) ist $k_{r}$ definiert als die Geschwindigkeitskonstante der strahlenden Deaktivierung des emittierenden ^3^MLCT‐Zustands, $k_{\mathrm{ISC}}$ als die Intersystem‐Crossing (ISC)‐Rate vom emittierenden Zustand zum Grundzustand $S_{0}$ und $k_{\mathrm{nr}}$ als die thermisch aktivierte nicht‐radiative Rate, die durch die Arrhenius‐ähnliche Ratenkonstante ausgedrückt werden kann.

(2) $$k_{\mathrm{nr}}={\mathrm{Ae}}^{\frac{-\Delta E}{\mathrm{RT}}}$$

Zu beachten ist, dass die Ratenkonstanten k_r_ und k_nr_ Deaktivierungsprozesse beschreiben, welche vom gleichen elektronisch angeregten Zustand (^3^MLCT) ausgehen, aber über unterschiedliche Wege ablaufen. Der Term k_nr_ erlaubt es, die Emissionslebensdauern mit der Deaktivierung durch die nicht emittierenden ^3^MC‐Zustände zu korrelieren. Die bahnbrechenden Arbeiten von Van Houten und Watts,^[10]^ gefolgt von Caspar und Meyer,^[17]^ lieferten einige der ersten experimentellen Werte für ΔE , indem sie Gleichung 2 auf ein breites Spektrum von Temperaturen und Lösungsmitteln anwendeten. Im Anschluss an diese Arbeiten wurde ΔE seither entweder als die Energielücke (*g*) zwischen den niedrigsten ^3^MLCT‐ und ^3^MC‐Zuständen[^10^, ^18^, ^19^, ^20^, ^21^] oder als die Energiebarriere (*E_a_*) zwischen beiden Zuständen[^14^, ^17^, ^22^, ^23^, ^24^, ^25^, ^26^, ^27^, ^28^, ^29^, ^30^] (siehe Abbildung 1) interpretiert.

In dieser Arbeit demonstrieren wir, dass es sich bei diesen Interpretationen (in Verbindung mit dem vereinfachten Jablonski‐Diagramm von Abbildung 1) um eine Vereinfachung handelt, welche andere temperaturabhängige, nicht‐strahlende Pfade und die Rolle der Schnittpunkte zwischen den relevanten Zuständen vernachlässigt. Wir behaupten, dass die weit verbreitete Annahme, dass ΔE nur mit einem einzelnen Relaxationsprozess zusammenhängt, im Allgemeinen eine unscharfe Definition darstellt und zu falschen theoretischen Vorhersagen der Emissionslebensdauer führt. Bis heute werden solche inkorrekten Vorhersagen der Ungenauigkeit der verwendeten quantenchemischen Methode zugeschrieben, da sehr kleine Änderungen im Term ΔE der Gleichung 2 leicht zu ungenauen Reaktionsraten mit Ungenauigkeiten führen, die mehrere Größenordnungen umfassen können. Dies kann zu folgenden Missverständnissen führen: irreführende zufällige lineare Korrelationen zwischen der Aktivierungsenergie *E_a_* und der experimentell gemessenen Quanteneffizienz, falsche theoretische Benchmarks, und falsche Struktur‐Eigenschafts‐Beziehungen, bei denen Verbindungen mit zunehmenden ^3^MLCT‐^3^MC Energielücken optimiert werden, aber die Emissionslebensdauern nicht zwangsläufig zunehmen.

In dieser Arbeit zeigen wir, dass diese Unstimmigkeiten mit der Verwendung des falschen kinetischen Modells für die nicht‐radiative Relaxation von ${\left[\mathrm{Ru}{\left(\mathrm{bpy}\right)}_{3}\right]}^{2+}$ und mit der Anwendung des Konzepts des *ratenbestimmenden Schritts* anstelle des *ratenbestimmenden Zustands* zusammenhängen.[^31^, ^32^, ^33^, ^34^] Wir demonstrieren, dass die Deaktivierung von ${\left[\mathrm{Ru}{\left(\mathrm{bpy}\right)}_{3}\right]}^{2+}$ ein komplexerer Prozess ist, als bisher experimentell angenommen wurde.[^10^, ^17^] Auf Grundlage umfassender und genauer quantenchemischer Berechnungen des vollständigen energetischen Profils des emittierenden ^3^MLCT‐Zustands lassen sich bei Verwendung des richtigen kinetischen Modells Emissionslebensdauern berechnen. Die Gültigkeit unseres Ansatzes für ${\left[\mathrm{Ru}{\left(\mathrm{bpy}\right)}_{3}\right]}^{2+}$ wird durch Messungen der temperaturabhängigen Emissionslebensdauer bestätigt.

## Resultate und Diskussion

**Figure 2:**
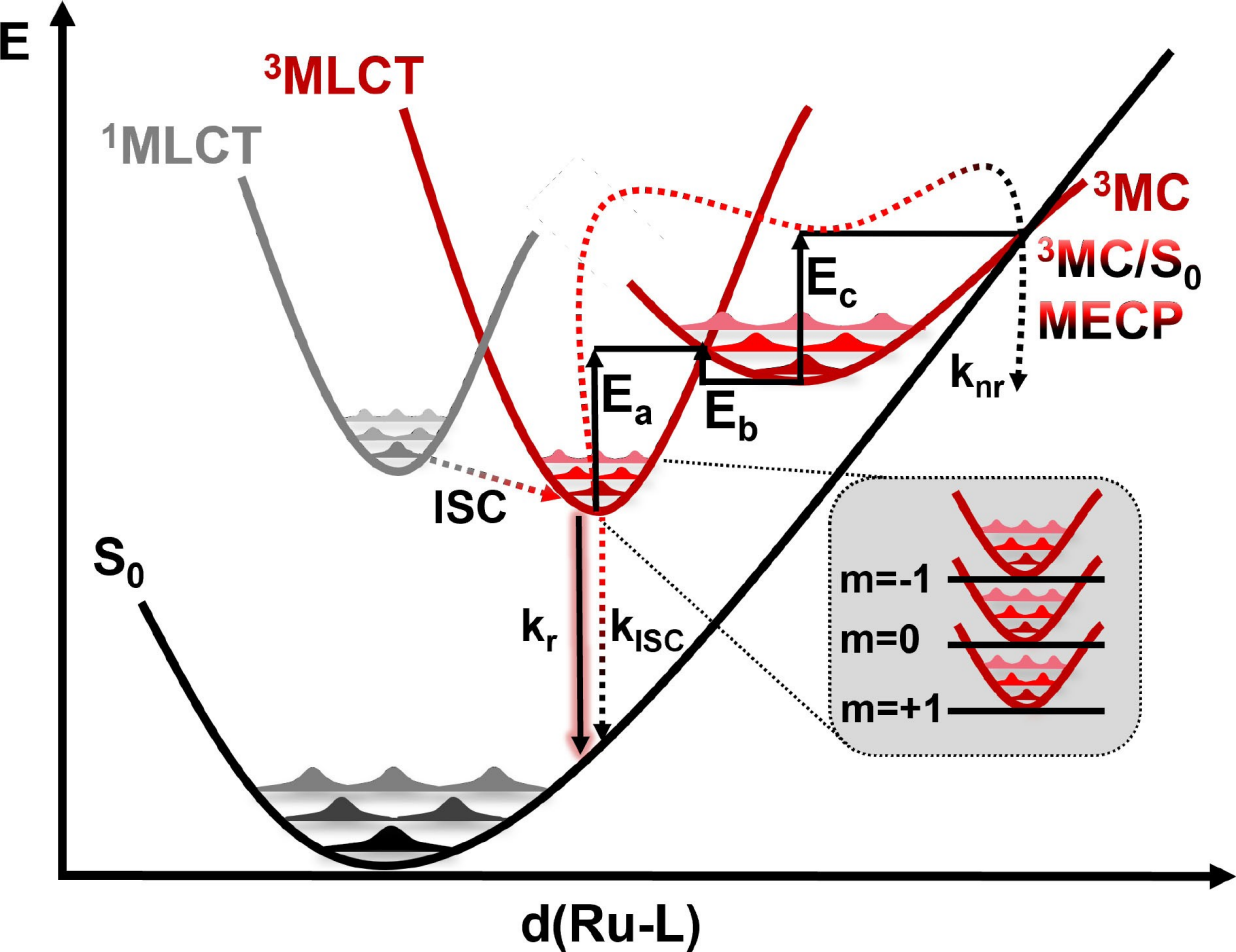
Potentielle Energieflächen, die den wesentlichen photophysikalischen Prozess für die Deaktivierung von ${\left[\mathrm{Ru}{\left(\mathrm{bpy}\right)}_{3}\right]}^{2+}$ und wahrscheinlich verwandten Ru‐basierten Komplexen darstellen. Zu beachten ist, dass knr die gesamte nicht‐radiative Rate vom 3MLCTZustand über den 3MC‐Zustand zum Grundzustand S0 bezeichnet.

Im Vergleich zu Abbildung 1 präsentiert Abbildung 2 ein umfassenderes Schema der wichtigsten photophysikalischen Prozesse, die der Deaktivierung von ${\left[\mathrm{Ru}{\left(\mathrm{bpy}\right)}_{3}\right]}^{2+}$ artigen Ru‐Metall‐Komplexen zugeschrieben werden. Bei Lichteinstrahlung bildet sich eine Vielzahl von Singulett‐^1^MLCT‐Zuständen aus. In ${\left[\mathrm{Ru}{\left(\mathrm{bpy}\right)}_{3}\right]}^{2+}$ erfolgt ISC zum Triplett‐Zustand ^3^MLCT in weniger als 30 fs,[^35^, ^36^, ^37^] gefolgt von einer internen Umwandlung in den niedrigsten ^3^MLCT‐Zustand, von dem aus die Phosphoreszenz mit der Geschwindigkeitskonstante $k_{r}$ stattfindet. Die Spin‐Bahn‐Kopplung hebt die dreifache Entartung der Triplett‐Zustände auf, so dass ein statistischer Boltzmann‐Ansatz der niedrigsten drei Spin‐Subniveaus für eine genaue Berechnung von $k_{r}$ erforderlich ist. Vom ^3^MLCT Zustand ist auch eine nicht‐strahlende Relaxation über ISC zum elektronischen Grundzustand S_0_ möglich. Aufgrund der Zunahme der Population hochenergetischer Schwingungszustände ist dieser Prozess temperaturabhängig und steht mit der Geschwindigkeitskonstante $k_{\mathrm{ISC}}$ in Verbindung. Bei Erhöhung der Temperatur können auch ^3^MC‐Zustände populiert werden, was zu einer irreversiblen nicht‐strahlenden Deaktivierung ($k_{\mathrm{nr}}$ ) zum S_0_ über den ^3^MC/S_0_‐Minimum Energy Crossing Point (MECP) führt. Vor allem die zunehmende Besetzung der niedrigsten ^3^MC‐Zustände bei Raum‐ oder höherer Temperatur führt zu einer Verkürzung der Emissionslebensdauer.^[17]^

**Figure 3:**
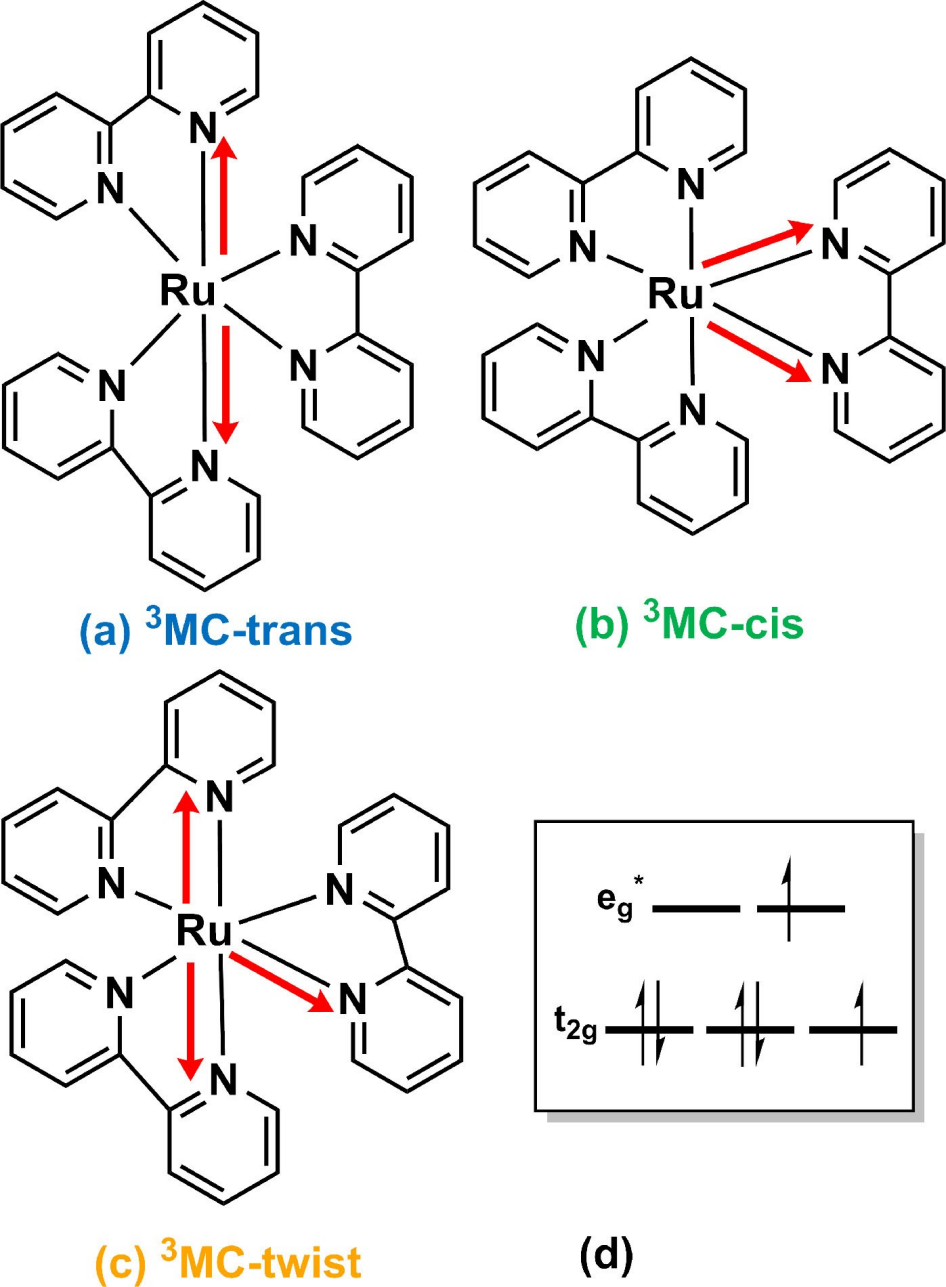
Trans (a), cis (b) und twist (c) Jahn–Teller‐Isomere von ${\left[\mathrm{Ru}{\left(\mathrm{bpy}\right)}_{3}\right]}^{2+}$ mit ^3^MC‐Charakter, wie in früheren Studien beschrieben.[^27^, ^40^, ^42^, ^43^] Die roten Pfeile beschreiben die charakteristischen verlängerten Ru−N‐Bindungen, die aus der Population der σ ‐antibindenden $e_{g}^{*}$ Orbitale (d) stammen.

Aufgrund ihrer Kurzlebigkeit ist die experimentelle Strukturaufklärung der ^3^MC‐Zustände eine Herausforderung.[^25^, ^38^, ^39^] Der erste Vorschlag für eine ^3^MC‐Geometrie in ${\left[\mathrm{Ru}{\left(\mathrm{bpy}\right)}_{3}\right]}^{2+}$ stammt, nach unserem Wissensstand, aus der theoretischen Arbeit von Alary et al.^[40]^ Die Autoren schlugen eine Geometrie mit zwei verlängerten axialen Ru−N‐Bindungen^[40]^ vor. Diese ist auf eine Jahn–Teller^[41]^ (JT) verzerrte Struktur zurückzuführen, bei welcher sich die Symmetrie von D_3_ auf C_2_ verringert. Diese Struktur wird hier als ^3^MC‐trans JT‐Isomer bezeichnet (Abbildung 3a). Das Vorhandensein kleiner imaginärer Frequenzen deutete darauf hin, dass es sich nicht um ein echtes Minimum handelte, und obwohl die Berechnungen fälschlicherweise diese Geometrie energetisch niedriger als den emittierenden ^3^MLCT‐Zustand einstuften,^[40]^ wurde sie lange Zeit als die Schlüsselstruktur zur Erklärung der nicht‐strahlenden Relaxation von ${\left[\mathrm{Ru}{\left(\mathrm{bpy}\right)}_{3}\right]}^{2+}$ und vielen homoleptischen 2,2’‐Bipyridin‐Analoga erachtet. Ein Jahrzehnt später wurde die Struktur mit genaueren theoretischen Methoden erneut optimiert und das wahre ^3^MC‐trans‐Minimum identifiziert.^[27]^ In Übereinstimmung mit den experimentellen Erwartungen lag dessen Energie über der des ^3^MLCT‐Zustands.[^10^, ^17^, ^38^] Interessanterweise hatten dieselben Autoren auch zwei weitere Minima mit ^3^MC‐Charakter bestimmt. Eines^[42]^ ist durch zwei verlängerte Ru−N‐Bindungen zu demselben Bipyridylliganden charakterisiert. Diese Struktur wird hier als das cis‐JT‐Isomer bezeichnet (Abbildung 3b). Das andere Minimum^[43]^ zeichnet sich durch zwei verlängerte Ru−N‐Bindungen in zwei verschiedenen Bipyridylliganden und einen N−Ru−N ‐Winkel (der jeweiligen verlängerten Ru−N‐Bindungen) von nahezu 90 Grad aus. Daher bezeichnen wir es als ^3^MC‐twist JT‐Isomer (Abbildung 3c). Zu beachten ist, dass die beiden letztgenannten Geometrien in der Literatur nur im Zusammenhang mit der lichtinduzierten Abspaltung des/der Bipyridin‐Liganden diskutiert wurden,[^42^, ^43^] aber nie zur Berechnung von Lumineszenzlebensdauern berücksichtigt wurden.

Im Folgenden werden wir zeigen, wie das Schicksal des ^3^MLCT‐Zustands weniger vom ^3^MC‐trans‐Minimum und mehr von den cis‐ und‐twist‐JT‐Isomeren abhängt, welche bisher vernachlässigt wurden.

**Figure 4:**
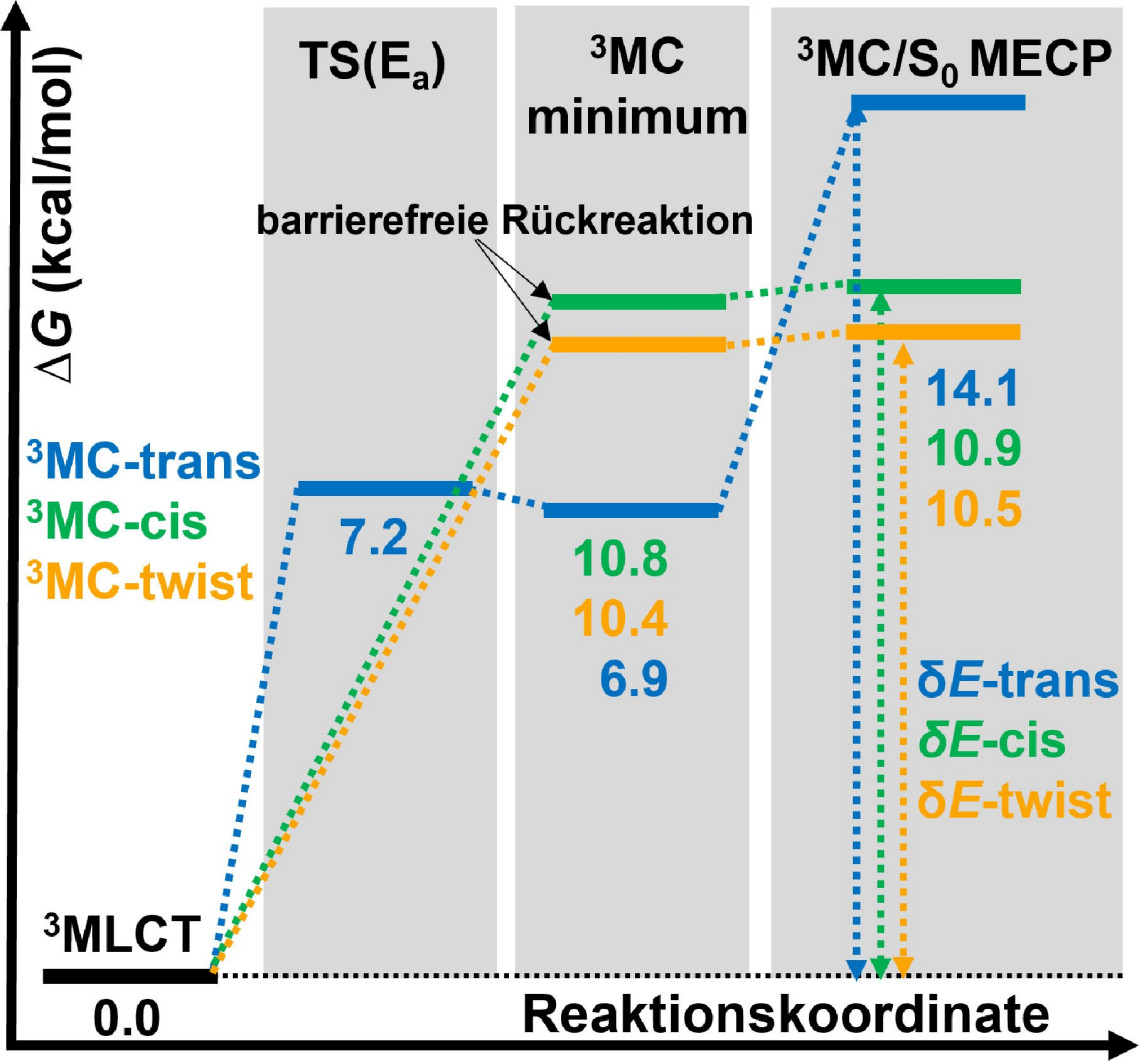
Berechnete relative freie Gibbs‐Energien bei 298,15 K für den nicht‐radiativen Zerfall des niedrigsten ^3^MLCT‐Zustands durch die JT‐Isomere ^3^MC‐trans (blau), ^3^MC‐cis (grün) und ^3^MC‐twist (orange). *δE* ist die Energiespanne/effektive Reaktionsbarriere für die jeweiligen Pfade (siehe Diskussion im Text). Die Energien sind in kcal/mol angegeben, bezogen auf den ^3^MLCT‐Zustand (0,0 kcal/mol). Quantenchemische Methode: B2GP‐PLYP−D3/def2‐QZVPP@CPCM(Acetonitril)//B3LYP−D3/def2‐SVP@CPCM(Acetonitril)

Zunächst berechnen wir die relativen freien Energien für die Relaxation von ${\left[\mathrm{Ru}{\left(\mathrm{bpy}\right)}_{3}\right]}^{2+}$ unter Berücksichtigung der Isomere ^3^MC‐trans (blau in Abbildung 4), ^3^MC‐cis (grün) und ^3^MC‐twist (orange). Um genaue Vorhersagen anzustreben, verwenden wir ein double hybrid functional[^44^, ^45^, ^46^] und umfangreiche Basissätze (siehe Abschnitt S1 der unterstützenden Informationen für weitere Berechnungsdetails). Diese Kombination ermöglicht eine Genauigkeit, die über die des häufig verwendeten B3LYP‐Hybridfunktionals hinausgeht.[^27^, ^28^, ^42^]

Für das trans‐JT‐Isomer fanden wir, wie auch in früheren theoretischen Studien,[^27^, ^42^] einen Übergangszustand zwischen dem niedrigsten ^3^MLCT‐ und ^3^MC‐Zustand, dessen Energie 7,2 kcal/mol beträgt. Für die cis‐ und twist‐Strukturen, die in der Vergangenheit in diesem Zusammenhang nie untersucht wurden, fanden wir keine Übergangszustände, was darauf hindeutet, dass die letztgenannten Pfade barrierefrei sind (Abbildung 4). Die berechneten Energien der entsprechenden ^3^MC‐Minima für die trans‐, cis‐ und twist‐JT‐Isomere liegen bei 6,9, 10,8 und 10,4 kcal/mol. Mit diesen Informationen könnte man zu dem Schluss kommen, dass der Weg über das ^3^MC‐trans‐Isomer der einzige ist, der für die Beschreibung des Emissionsverhalten von ${\left[\mathrm{Ru}{\left(\mathrm{bpy}\right)}_{3}\right]}^{2+}$ relevant ist, da dieser die niedrigste ^3^MLCT→ ^3^MC‐Reaktionsbarriere (7,2 kcal/mol) im Vergleich zum cis‐Isomer (10,8 kcal/mol) und dem Twist‐Isomer (10,4 kcal/mol) aufweist. Beschränkt man sich jedoch auf diese Argumentation, so müsste die berechnete Reaktionsbarriere zum Erreichen des ^3^MC‐trans‐Minimums (7,2 kcal/mol) oder die Energielücke zwischen beiden Zuständen (6,9 kcal/mol) mit dem von Caspar und Meyer experimentell bestimmten ΔE Wert von 10,9 kcal/mol ($3800\;{\mathrm{cm}}^{-1}$ ) übereinstimmen.^[17]^ Dies ist jedoch nicht der Fall. Entsprechende Unstimmigkeiten wurden in der Vergangenheit mit der verwendeten quantenchemischen Methode und deren innewohnenden Fehlern begründet. In dieser Arbeit lassen wir dieses Argument außer Acht. Zum einen, aufgrund der hier verwendeten Methode und zusätzlich, und das ist am wichtigsten, weil es keinen experimentellen Beweis dafür gibt, dass der ermittelte Wert von 10,9 kcal/mol der Aktivierungsbarriere *E_a_* oder der Energielücke *g* in ${\left[\mathrm{Ru}{\left(\mathrm{bpy}\right)}_{3}\right]}^{2+}$ zuzuschreiben ist (vgl. Abbildung 1).

Um diese Interpretation in Frage zu stellen, betrachten wir die folgende Reaktion im Gleichgewichtszustand,
(3)

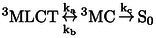




welche alle Schritte ausgehend vom ^3^MLCT Zustand über einen einzelnen ^3^MC Zustand beschreibt. Jede beteiligte Rate kann durch einen Wechsel von einer Energie‐ zu einer k‐Repräsentation unter Verwendung der Eyring‐Gleichung berechnet werden,

(4) $$k=\kappa \frac{k_{B}T}{h}e^{\frac{-\Delta G^{\neq }}{\mathrm{RT}}}$$

wobei $\Delta G^{\neq }$ die freie Gibbs'sche Aktivierungsenergie, $k_{B}$ die Boltzmann‐Konstante, h die Planck'sche Konstante und *κ* der Transmissionskoeffizient ist, der in der klassischen Formulierung der Übergangszustandstheorie auf 1 gesetzt wird.^[47]^ Für die in Gleichung 3 formulierte chemische Reaktion kann die Gesamtreaktionsrate $k_{\mathrm{nr}}$ dann wie folgt formuliert werden5

(5) $$k_{\mathrm{nr}}=\frac{k_{c}*k_{a}}{k_{b}+k_{c}}.$$

Im Falle, dass die Deaktivierung über den ^3^MC‐Zustand durch einen 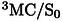 ‐MECP viel schneller abläuft als die Rückreaktion des ^3^MC‐Zustands zum ^3^MLCT‐Zustand, d. h. $k_{c}\gg k_{b}$ , dann würde $k_{\mathrm{nr}}=k_{a}$ gelten und die ^3^MLCT/^3^MC‐trans‐Reaktionsbarriere die Deaktivierung des emittierenden ^3^MLCT‐Zustands dominieren. Dieser Fall wird in der Literatur häufig für ${\left[\mathrm{Ru}{\left(\mathrm{bpy}\right)}_{3}\right]}^{2+}$ angenommen, was bedeutet, dass der 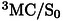 MECP energetisch unterhalb des Übergangszustands des ^3^MLCT→ ^3^MC‐Reaktionsschritts liegen sollte. Dies ist jedoch eine ad‐hoc‐Hypothese, die nicht durch das von uns (Abbildung 4) und anderen^[28]^ berechnete Reaktionsprofil gestützt wird. Stattdessen werden unsere Berechnungen zeigen, dass $k_{a}$ alleine nicht ausreichend ist, um die Gesamtrate der nicht‐strahlenden Relaxation durch den ^3^MC‐Zustand zu bestimmen, vor allem nicht, wenn $k_{a}$ von einem einzelnen Prozess bestimmt wird.

Da der ^3^MLCT/^3^MC‐Schritt (Gleichung 3) die kleinste Geschwindigkeitskonstante ($k_{a}$ ) aufweist, ist es nicht verwunderlich, dass bisher die ^3^MC‐Zustände verwendet wurden, um Struktur‐Eigenschafts‐Beziehungen herzuleiten, in der Hoffnung, dass dies Aufschlüsse für das Design langlebiger lumineszenter ^3^MLCT‐Zustände liefert. In diesem Bestreben wurden, möglicherweise aus historischen Gründen, lediglich ^3^MC‐Zustände mit einer trans‐Verzerrung berücksichtigt. Allerdings, wie in mehreren Publikationen hervorgehoben wurde,[^31^, ^32^, ^33^, ^34^] ist das Konzept eines *ratenbestimmenden Schritts* bei der Erklärung von Reaktionsmechanismen oft unzureichend und nicht eindeutig. Stattdessen wird die gesamte Ratenkontrolle eines bestimmten Prozesses durch Zwischenstufen und Übergangszustände bestimmt. Dieser werden als *ratenbestimmende Zustände* bezeichnet. Bei diesen handelt es sich um die Zustände mit den höchsten effektiven Energiebarrieren. In unserem Fall (Abbildung 4) sollte die effektive Energiebarriere durch die energetische Differenz zwischen dem ^3^MLCT‐Minimum und dem 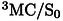 ‐MECP gegeben sein, eine Größe, die für jeden der ^3^MC‐trans‐, cis‐ und twist‐Reaktionspfade definiert werden kann. Diese effektive Energiebarriere wird als *energetische Spanne* (*δE*) bezeichnet, weshalb dieses Konzept in der Literatur als *Energetisches Spannmodel* bekannt ist.[^31^, ^32^, ^33^, ^34^]

Unter Berücksichtigung dieses Modells, ist zu erwarten, dass der experimentell bestimmte^[17]^ ΔE ‐Wert von 10,9 kcal/mol (Gleichung 1) der *energetischen Spanne* zwischen dem ^3^MLCT‐Zustand und dem 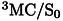 ‐MECP entspricht. Eine einfache Methode zur Identifizierung solcher geschwindigkeitsbestimmenden Zustände ist die Simulation des Grades, mit dem Zwischenstufen und Übergangszustände/Schnittpunkte die Reaktionsrate beeinflussen.[^48^, ^49^] Durch Beobachtung der Änderungen der Gesamtreaktionsrate bei differentiellen Änderungen der relativen Energien der einzelnen am Mechanismus beteiligten Zustände kann diese Methode realisiert werden (Abschnitt S2). Unseren Berechnungen zufolge (Abbildung 4) beinhaltet die Relaxation über den ^3^MC‐Zustand *energetische Spannen δE* von 14,5 kcal/mol, 10,9 kcal/mol bzw. 10,5 kcal/mol für die trans‐, cis‐ und twist‐JT‐Isomere. Sowohl die cis‐ als auch die twist‐effektiven Energiebarrieren sind, innerhalb des Fehlers der Methode, in Übereinstimmung mit dem gemessenen ΔE ,^[17]^ während die trans‐Energie große Diskrepanzen aufweist. Hervorzuheben ist, dass wir bereits jetzt feststellen können, dass (i) ΔE weder mit der Energiebarriere *E_a_* vom ^3^MLCT‐ zu einem ^3^MC‐Zustand noch mit der Energielücke *g* zwischen beiden Zuständen in Verbindung gebracht werden sollte, sondern mit der *energetischen Spanne*, und (ii), dass der typische ^3^MC‐trans‐Relaxationsweg nicht relevant für das Schicksal des emittierenden ^3^MLCT‐Zustands ist, sondern sowohl der ^3^MC‐cis‐ als auch der ‐twist‐Reaktionsweg zur Relaxation von ${\left[\mathrm{Ru}{\left(\mathrm{bpy}\right)}_{3}\right]}^{2+}$ und damit zur Lebensdauer des emittierenden ^3^MLCT‐Zustands beitragen. An dieser Stelle sei angemerkt, dass Escudero^[50]^ vorschlug, den Begriff ΔE in Gleichung 2 als $E_{\lim}$ zu quantifizieren, welcher, wie in seiner Arbeit mathematisch definiert, der *energetischen Spanne δE* hätte entsprechen sollen, er definierte ihn jedoch als “die Aktivierungsenergie für den limitierenden Schritt”, was zu irreführenden Verwendungen geführt hat.^[51]^

Mit unserem neuen Protokoll wollen wir nun das aus den quantenchemischen Berechnungen gewonnene Energieprofil mit gemessenen temperaturabhängigen Emissionslebensdauern in Verbindung bringen. Berechnungen der temperaturabhängigen photolumineszenten Eigenschaften sind bis auf wenige Ausnahmen, wie die Arbeit von Escudero und Mitarbeitern an drei Ir(III)‐Komplexen^[52]^ die Ähnlichkeiten in ihrem Lumineszenzabfall mit ${\left[\mathrm{Ru}{\left(\mathrm{bpy}\right)}_{3}\right]}^{2+}$ aufweisen, selten. Die Autoren waren in der Lage, alle Terme der Gleichung 1 zu berechnen, erhielten aber nur eine qualitative Übereinstimmung mit experimentellen Daten, da die Emissionsquantenausbeuten in allen Fällen um Faktoren zwischen 1.35 und 11 unterschätzt wurden.^[52]^

Frühe experimentelle Emissionslebensdauern von ${\left[\mathrm{Ru}{\left(\mathrm{bpy}\right)}_{3}\right]}^{2+}$ , aus denen der ΔE Wert von 10,9 kcal/mol bestimmt wurde, wurden in Acetonitril von 77 bis 300 K aufgezeichnet.^[17]^ In diesem Niedrigtemperaturbereich ist bekannt,^[52]^ dass die Temperaturabhängigkeit von $k_{\mathrm{ISC}}$ und $k_{r}$ dominiert, während bei höheren Temperaturen $k_{\mathrm{nr}}$ dominiert. Dies bedeutet, dass die experimentelle Auswertung von ΔE über Gleichung 1 umso genauer ist, je höher die Temperatur ist. Dies veranlasste uns, die experimentellen Daten für ${\left[\mathrm{Ru}{\left(\mathrm{bpy}\right)}_{3}\right]}^{2+}$ in Acetonitril im Bereich von 280 K–350 K zu überprüfen (siehe experimentelle Details in Abschnitt S3).

**Figure 5:**
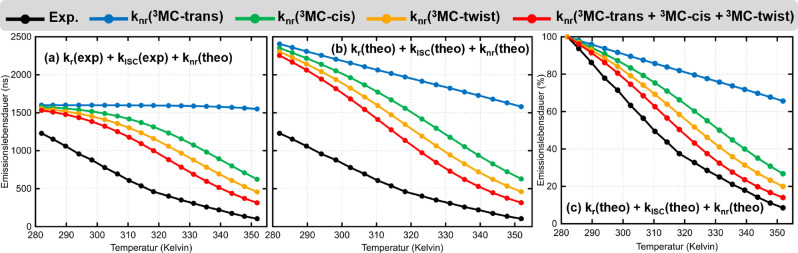
Temperaturabhängige Emissionslebensdauern von [Ru(bpy)_3_]^2+^ in ns (a und b) und in Prozent (c). Die schwarze Linie gibt die experimentellen Werte an. Die blauen, grünen und orangefarbenen Linien sind die rechnerisch ermittelten Lebensdauern unter Verwendung der nicht‐radiativen Raten k_nr_ der Isomere ^3^MC‐trans, ^3^MC‐cis bzw. ^3^MC‐twist. Die rote Linie berücksichtigt alle drei Pfade, wie in Gleichung 6 beschrieben. Grafik (a) zeigt berechnete Ergebnisse, die mit experimentellen k_r_‐ und k_ISC_‐Werten erhalten wurden. Die Grafiken (b) und (c) verwenden theoretisch berechnete k_r_‐ und k_ISC_‐Werte.

Die experimentellen temperaturabhängigen Emissionslebensdauern sind in Abbildung 5a (schwarze Linie) aufgetragen. Diese Werte werden mit den nach Gleichung 1 berechneten Lebensdauern verglichen. Für die Geschwindigkeitskonstanten $k_{r}$ und $k_{\mathrm{ISC}}$ verwenden wir zunächst experimentell geschätzte Werte (Abschnitt S3), so dass sich der Vergleich zwischen Experiment und Theorie auf die rechnerische Genauigkeit von $k_{\mathrm{nr}}$ (dominant bei hohen Temperaturen) konzentrieren kann. Für $k_{\mathrm{nr}}$ verwenden wir Gleichung 5, wobei die Ratenkonstanten $k_{a,b,c}$ aus den freien Gibbs‐Aktivierungsenergien $\Delta G_{a,b,c}^{\neq }$ berechnet werden (siehe Abbildung 2). Die Verwendung der quantenchemischen Werte, die für die ^3^MC‐trans‐, ^3^MC‐cis‐ oder ^3^MC‐twist‐Pfade (Abschnitt S2, Tabelle S8) berechnet wurden, liefert die blauen, grünen und orangen Plots in Abbildung 5a.

Die Kurven, die mit den ^3^MC‐cis und ^3^MC‐twist JT‐Isomeren berechnet wurden, zeigen eine sehr gute Übereinstimmung mit dem Experiment. Darüber hinaus bestätigt die Tatsache, dass die Kurven für beide JT‐Isomere im Wesentlichen identisch sind, dass beide Wege von vergleichbarer Gewichtung sind. Ein übereinstimmendes Verhalten der beiden Isomere und ihr unterschiedliches Verhalten im Vergleich zum ^3^MC‐trans‐Isomer lässt sich durch den Charakter der besetzten $e_{g}^{*}$ ‐Orbitale am Ruthenium‐Metallzentrum erklären. Das ^3^MC‐trans‐Isomer zeigt die Besetzung eines $d_{z^{2}}$ ‐ähnliches dσ*‐Orbitals, während die ^3^MC‐cis‐ und ^3^MC‐twist‐JT‐Isomere ein besetztes $d_{x^{2}-y^{2}}$ ‐ähnliches dσ*‐Orbital aufweisen (siehe Abbildung S3).[^27^, ^42^, ^43^] Der Charakter der besetzten antibindenden Orbitale, wenn man vom emittierenden ^3^MLCT zu einem Metall‐zentrierten angeregten Zustand übergeht, ist ebenfalls konsistent mit der entsprechenden verzerrten Ru−N‐Bindung (Abbildung S3). Im Gegensatz dazu zeigen die Berechnungen deutlich, dass die Emissionslebensdauer bei höheren Temperaturen nicht durch den Weg über das ^3^MC‐trans JT‐Isomer bestimmt wird (blaue Linie). Dies steht in starkem Widerspruch zu früheren theoretischen Studien über Ruthenium‐[^14^, ^53^, ^54^, ^55^, ^56^, ^57^, ^58^, ^59^] und Iridium‐[^11^, ^50^, ^52^, ^60^, ^61^] Komplexe, die sich auf ein Isomer konzentrieren, entweder das prototypische trans‐Isomer oder nur den niedrigstliegenden MC‐Zustand, um experimentelle Daten zu vergleichen oder zu erklären.

Aus unseren Ergebnissen schlussfolgern wir, dass das experimentell gemessene $k_{\mathrm{nr}}$ aus einer gleichwertigen Betrachtung aller Deaktivierungspfade hervorgehen sollte. Daher berechnen wir die Emissionslebensdauer als,6

(6) $$\tau_{\mathrm{Emission}}^{'}=\frac{1}{k_{r}{+k}_{\mathrm{ISC}}{+k}_{\mathrm{nr}}{(}^{3}\mathrm{MC}-\mathrm{trans}){+k}_{\mathrm{nr}}{(}^{3}\mathrm{MC}-\mathrm{cis}){+k}_{\mathrm{nr}}{(}^{3}\mathrm{MC}-\mathrm{twist})}$$

wobei $k_{\mathrm{nr}}{(}^{3}\mathrm{MC}-\mathrm{trans})$ , $k_{\mathrm{nr}}{(}^{3}\mathrm{MC}-\mathrm{cis})$ und $k_{\mathrm{nr}}{(}^{3}\mathrm{MC}-\mathrm{twist})$ die nicht‐strahlenden Relaxationsraten durch die ^3^MC‐trans, ^3^MC‐cis bzw. ^3^MC‐twist Pfade beschreiben. Das erhaltene Ergebnis (rote Kurve in Abbildung 5a) verbessert die Übereinstimmung mit den experimentellen Daten zusätzlich und bestätigt die Gültigkeit der erweiterten Gleichung 6. Verbleibende kleine Abweichungen (z. B. die Position des konkav‐konvexen Knickpunkts) können auf die verwendete quantenchemische Methode und möglicherweise auf die Tatsache zurückgeführt werden, dass wir einen konstanten Wert für $k_{\mathrm{ISC}}$ und $k_{r}$ annehmen, der auf der Basis von experimentellen Messungen geschätzt wurde. Wir weisen darauf hin, dass eine Abweichung von 1 kcal/mol der geschätzten Energiebarrieren zu einer Abweichung der Raten von einer Größenordnung führen kann (Gleichung 4), so dass wir mit den derzeitigen elektronischen Strukturmethoden vermutlich das Limit erreicht haben.

Eine Verbesserung unserer theoretischen Schätzungen der Lebensdauern könnte durch die Einbeziehung der temperaturabhängigen $k_{\mathrm{ISC}}$ ‐ und $k_{r}$ ‐Raten ermöglicht werden. Dementsprechend berechnen wir $k_{\mathrm{ISC}}$ und $k_{r}$ (Abschnitt S1), um die Emissionslebensdauer in einem vollständig theoretischen Rahmen zu bewerten. Die Ergebnisse, die in Abbildung 5b (für die einzelnen JT‐Isomere und die Summe) dargestellt sind, ähneln denen von Abbildung 5a, d. h. bei höheren Temperaturen kann der Weg über das trans‐JT‐Isomer die Emissionslebensdauern nicht reproduzieren; vielmehr sind die Wege über die cis‐ und twist‐JT‐Isomere für die Relaxation verantwortlich. Die Übereinstimmung ist bei höheren Temperaturen deutlich besser, was auf unsere erweiterte Beschreibung der Rate $k_{\mathrm{nr}}$ zurückzuführen ist, welche folglich zum dominierenden Pfad der Relaxation wird. Die Diskrepanzen bei niedrigen Temperaturen sind wahrscheinlich auf die harmonische Näherung bei der Berechnung von $k_{\mathrm{ISC}}$ zurückzuführen. Zum Vergleich: die Emissionslebensdauer, die bei der Verwendung von $k_{a}$ (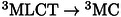 ) anstelle von $k_{\mathrm{nr}}$ (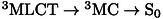 ) durch die Gleichung 6 bestimmt wird, ist nahezu Null (Abbildung S5). Dieses Ergebnis unterstreicht die Konsequenzen, die mit einer vereinfachten Formulierung der nicht‐strahlenden Relaxationsrate des angeregten Zustands ^3^MLCT verbunden sind (siehe weitere Diskussion in Abschnitt S2.4). Abbildung 5c zeigt die Emissionslebensdauern als prozentuale Darstellung, wobei die Werte aus Tafel b übernommen wurden. Auch hier ist eine hohe Übereinstimmung mit dem Experiment festzustellen, welche der Einbeziehung aller JT‐Isomere in die Berechnung der nicht‐radiativen Konstante zugrunde liegt. Im Gegensatz dazu kann das trans‐JT‐Isomer allein das Temperaturverhalten nicht erklären, was sich mit zunehmender Temperatur verschlechtert.

## Zusammenfassung

**Figure 6:**
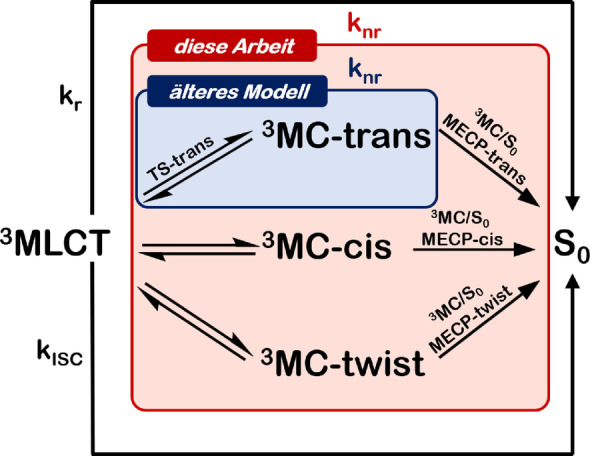
Überblick über die in der Vergangenheit und in dieser Arbeit vorgeschlagenen kinetischen Modelle für ${\left[\mathrm{Ru}{\left(\mathrm{bpy}\right)}_{3}\right]}^{2+}$ .

Wir demonstrieren, dass die weit verbreitete Ansicht, dass Emissionslebensdauern von ${\left[\mathrm{Ru}{\left(\mathrm{bpy}\right)}_{3}\right]}^{2+}$ und ähnlichen Verbindungen durch Beeinflussung der Energiebarriere zwischen dem emittierenden ^3^MLCT und dem thermisch aktiviertem ^3^MC Zustand, oder der Energiedifferenz zwischen den beiden Zuständen, kontrolliert werden könnten, im Allgemeinen nicht zutrifft. Darüber hinaus ist der Fokus auf den geschwindigkeitsbestimmenden Schritt und damit auf einen einzelnen Relaxationspfad eine Vereinfachung, die zu falschen Vorhersagen der Lebensdauern in ${\left[\mathrm{Ru}{\left(\mathrm{bpy}\right)}_{3}\right]}^{2+}$ führen kann. Für diesen Komplex bedeutet es, dass die isolierte Betrachtung des Relaxationsweges, der mit dem niedrigsten ^3^MC‐Minimum (dem verzerrten trans‐JT‐Isomer) zusammenhängt, Emissionslebensdauern liefert, die mit zunehmender Temperatur signifikant vom Experiment abweichen, wenn die Reaktionsraten der nicht‐strahlenden Pfade dominieren. Stattdessen möchten wir hier aufzeigen, dass es möglich ist, quasi‐quantitative temperaturabhängige Lebensdauern zu berechnen, wenn die Pfade, die mit allen JT‐Isomeren verbunden sind, berücksichtigt werden und das Konzept der ratenbestimmenden Zustände verwendet wird. Beide kinetischen Modelle sind in Abbildung 6 zusammengefasst. Diese zeigt den Unterschied in der Beschreibung der nicht‐radiativen Rate durch die Metall‐zentrierten angeregten Zustände, welche entweder nur das ^3^MC‐trans‐Isomer (älteres Modell) oder alle JT‐Isomere (diese Arbeit) berücksichtigt. Da die Verwendung eines einzelnen Reaktionswegs (d. h. einer einzelnen 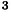 MC‐Geometrie) und/oder des niedrigsten 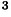 MC‐angeregten Zustands zur Berechnung der Lumineszenzlebensdauer in der Literatur sehr stark verwurzelt ist (ratenlimitierender Schritt), hoffen wir, dass diese Arbeit nicht nur zur Vorsicht rät, sondern auch zu einer Neubewertung der mechanistischen Interpretationen der photophysikalischen/photochemischen Prozesse in Übergangsmetallkomplexen, an denen 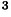 MC‐Zustände beteiligt sind, anregt.

Die Übertragung dieser Erkenntnisse von ${\left[\mathrm{Ru}{\left(\mathrm{bpy}\right)}_{3}\right]}^{2+}$ auf andere lumineszierende Übergangsmetallkomplexe ist unerlässlich für die Entwicklung von Verbindungen mit individuell angepassten Emissionslebensdauern. Je nach spezifischer Verbindung können die relative Position der ^3^MC‐Minima und ihre Deaktivierung in Richtung der ^3^MLCT‐Minima und des Grundzustands unterschiedlich sein, was sich auf das Gleichgewicht zwischen den verschiedenen JT‐Isomeren auswirkt.

## Beiträge

DHC: Methodologie, Berechnungen, Analyse und Verfassen des ursprünglichen Entwurfs. REPN: Synthese, Aufbereitung, Charakterisierung und Lebensdauermessungen. MAS: Analyse der experimentellen Daten. ST: Experimentelles Design. SR: Betreuung der experimentellen Arbeit, Bearbeitung des Manuskripts und Beschaffung von Geldern. LG: Konzeptualisierung, Analyse, Betreuung der theoretischen Arbeit, Überprüfung und Bearbeitung des Manuskripts, Administration des Projektes und Beschaffung von Geldern. Alle Autoren haben das Manuskript gelesen und stimmen ihm zu.

## Interessenkonflikt

Es liegen keine Interessenkonflikte vor.

1

## Supporting information

As a service to our authors and readers, this journal provides supporting information supplied by the authors. Such materials are peer reviewed and may be re‐organized for online delivery, but are not copy‐edited or typeset. Technical support issues arising from supporting information (other than missing files) should be addressed to the authors.

Supporting Information

Supporting Information

## Data Availability

Die Daten, die die Beobachtungen dieser Untersuchung unterstützen, sind auf Anfrage bei den korrespondierenden Autoren erhältlich.
