# Supplementary material for: Mehrere Triplett‐Metall‐zentrierte Jahn–Teller‐Isomere bestimmen die temperaturabhängigen Lumineszenzlebensdauern in [Ru(bpy)3]2+
Source: Angew Chem Weinheim Bergstr Ger. 2023 Sep 15;135(48):e202308803. doi: 10.1002/ange.202308803 (PMC10962581; doi:10.1002/ange.202308803)
Supplement: Supplementary file 2 — Supporting Information [file ANGE-135-0-s002.pdf]

Angewandte Chemie
Eine Zeitschrift der Gesellschaft Deutscher Chemiker

Supporting Information

Mehrere Triplett-Metall-zentrierte Jahn–Teller-Isomere bestimmen die temperaturabhängigen Lumineszenzlebensdauern in $[Ru(bpy)_3]^{2+}$

*D. Hernández-Castillo, R. E. P. Nau, M.-A. Schmid, S. Tschierlei, S. Rau, L. González**

Supporting Information

Multiple Triplet Metal-Centered Jahn-Teller Isomers Determine Temperature-Dependent Luminescence Lifetimes in $[Ru(bpy)_3]^{2+}$

David Hernández-Castillo,[†,‡] Roland E. P. Nau,[¶] Marie-Ann Schmid,[§] Stefanie Tschierlei,[§] Sven Rau,[¶] and Leticia González[*,†,‖]

†*Institute of Theoretical Chemistry, Faculty of Chemistry, University of Vienna, Währingerstr. 19, 1090 Vienna, Austria*

‡*Doctoral School in Chemistry (DoSChem), University of Vienna, Währinger Straße 42, 1090 Vienna, Austria*

¶*Institute of Inorganic Chemistry I, Ulm University, Albert-Einstein-Allee 11, 89081 Ulm, Germany*

§*Department of Energy Conversion, Institute of Physical and Theoretical Chemistry Technische Universität Braunschweig, Rebenring 31, 38106 Braunschweig, Germany*

‖*Vienna Research Platform on Accelerating Photoreaction Discovery, University of Vienna, Währingerstr. 19, 1090 Vienna, Austria*

E-mail: leticia.gonzalez@univie.ac.at

This document contains theoretical and experimental details, as well as additional data and analysis. XYZ coordinates of all geometries are provided in the additional file coordi-

nates.zip.

Contents

S1 Computational details **S7**

S1.1 Calculation of the non-radiative decay of $[Ru(bpy)_3]^{2+}$ S7

S1.1.1 Nudge elastic band calculations with Pysisyphus S8

S1.2 Calculation of the radiative rate S10

S1.3 Calculation of the intersystem crossing rate S11

S2 Further computational results **S11**

S2.1 Geometric features of the computed structures S11

S2.2 Highest energy Single-Occupied Molecular Orbitals. S13

S2.3 Simulating the "degree of rate control" with Concvar S14

S2.4 Temperature-dependent emission lifetimes. S15

S2.5 Computed Gibbs free energies for the ^{3}MC-trans, ^{3}MC-cis and ^{3}MC-twist isomers, over a range of temperatures. S17

S2.6 Computed non-radiative (k_{nr}) rates. S25

S2.7 Computed radiative (k_r) and intersystem-crossing (k_{ISC}) rates S26

S3 Experimental Details **S28**

S3.1 Synthesis of $[Ru(bpy)_3](PF_6)_2$ S28

S3.2 NMR spectroscopy S28

S3.3 Time-resolved spectroscopy S29

S3.4 Emission lifetime data and statistic data fit S31

S3.5 Arrhenius-like fit of temperature dependent lifetimes S32

References **S34**

List of Tables

S1 Relevant bond distances of all molecules computed in this work, following the numeration shown in Figure S2 . . . . S12

S2 Relative energies with respect to the (^{3}MLCT) state (0.0 kcal/mol) for the decay through the ^{3}MC-trans at different temperatures, at the B3LYP-D3/def2-QZVPP@CPCM(acetonitrile)//B3LYP-D3/def2-SVP@CPCM(acetonitrile) level of theory. S19

S3 Relative energies respect to the (^{3}MLCT) state (0.0 kcal/mol) for the decay through the ^{3}MC-cis, at different temperatures, at the B3LYP-D3/def2-QZVPP@CPCM(acetonitrile)//B3LYP-D3/def2-SVP@CPCM(acetonitrile) level of theory. S20

S4 Relative energies with respect to the (^{3}MLCT) state (0.0 kcal/mol) for the decay through the ^{3}MC-twist, at different temperatures, at the B3LYP-D3/def2-QZVPP@CPCM(acetonitrile)//B3LYP-D3/def2-SVP@CPCM(acetonitrile) level of theory. S21

S5 Relative energies with respect to the (^{3}MLCT) state (0.0 kcal/mol) for the decay through the ^{3}MC-trans, at different temperatures, at the B2GP-PLYP-D3/def2-QZVPP@CPCM(acetonitrile)//B3LYP-D3/def2-SVP@CPCM(acetonitrile) level of theory. S22

S6 Relative energies with respect to the (^{3}MLCT) state (0.0 kcal/mol) for the decay through the ^{3}MC-cis, at different temperatures, at the B2GP-PLYP-D3/def2-QZVPP@CPCM(acetonitrile)//B3LYP-D3/def2-SVP@CPCM(acetonitrile) level of theory. S23

S7 Relative energies with respect to the (^{3}MLCT) state (0.0 kcal/mol) for the decay through the ^{3}MC-twist, at different temperatures, at the B2GP-PLYP-D3/def2-QZVPP@CPCM(acetonitrile)//B3LYP-D3/def2-SVP@CPCM(acetonitrile) level of theory. S24

S8 Computed non-radiative rates for the decay through the ^{3}MC-trans, ^{3}MC-cis and ^{3}MC-twist states at different temperatures, at the B2GP-PLYP-D3/def2-QZVPP@CPCM(acetonitrile)//B3LYP-D3/def2-SVP@CPCM(acetonitrile) level of theory. S25
S9 Computed radiative (k_r) and intersystem-crossing (k_{ISC}) rates from the ^{3}MLCT state to the ground state, at different temperatures. S26
S10 Temperature calibration experiment for ACN in the lifetime measured set up. S30
S11 Maximums peaks for the absorption and emission spectra S30
S12 Emission lifetimes values as obtained by the fits perform in the studied temperature range. S32

List of Figures

S1 Nudge Elastic Band simulation of the ^{3}MLCT → ^{3}MC-trans, ^{3}MLCT → ^{3}MC-cis and ^{3}MLCT → ^{3}MC-twist pathways. S10
S2 Simplified structure of $[Ru(bpy)_3]^{2+}$ with numbered bond distances (Ru-N) that are reported in Table S1. . . . . S11
S3 Isosurface plots of the highest energy Singly Occupied Molecular Orbital of relevant structures, as indicated. S13
S4 Concentration variation of the ^{3}MLCT, as simulated with Concvar. In dash red are the results from the computed energy profile for the ^{3}MC-trans JT isomer, which we take as a reference, in green the results for a 1 kcal/mol increase in the Gibbs free energy reaction barrier of the TS-trans and in blue for a 1 kcal/mol increase in the Gibbs free energy ^{3}MC/^{1}S$_0$ MECP-trans. . . S15

S5 Temperature-dependent emission lifetimes of $[Ru(bpy)_3]^{2+}$ in ns. Experimental values are given by the black line. Blue, green and orange are the computational lifetimes obtained using the non-radiative rates k_{nr} (solid lines) or k_a (discontinuous lines) of the ^{3}MC-trans, ^{3}MC-cis or ^{3}MC-twist isomers, respectively. Red solid line considers all three pathways, as in equation 6 of the manuscript (discontinuous lines for k_a). Panel (a) shows computational results obtained using experimental k_r and k_{ISC} values. Panel (b) uses theoretically calculated k_r and k_{ISC} values. *Lifetimes when considering only the decay through the ^{3}MC-trans and with a rate quantified by k_a (blue discontinuous line), are not visible because they overlap with the discontinuous red line. . . S17
S6 On the top, computed reorganization energies projected over the normal modes of the ^{3}MLCT of $[Ru(bpy)_3]^{2+}$ and on the bottom, displacement vectors of the three dominant vibrations. S27
S7 Exponential lifetime fit on $[Ru(bpy)_3]^{2+}$ in inert acetonitrile by 8.8 °C (left) and 78.7 °C (right) with fitting residuals. S31
S8 Fitting of the excited-state lifetime with temperature following equation 5 . . S33

S1 Computational details

S1.1 Calculation of the non-radiative decay of $[Ru(bpy)_3]^{2+}$

For the description of the reaction pathway related to k_{nr}, the triplet geometries have been optimized with an unrestricted Kohn-Sham formalism using the B3LYP[1,2] hybrid density functional, in combination with the def2-SVP[3] basis set (def2-ECP[4] for the ruthenium atom) and the Grimme's D3 dispersion corrections.[5] The optimizations are done considering implicit solvent effects through the conductor-like polarizable continuum model (CPCM),[6,7] taking acetonitrile as solvent, as in the experimental measurements. The nature of all stationary points (minima and transition states) was verified through the analysis of calculated harmonic vibrational frequencies. Accurate Gibbs free energies have been obtained by adding thermodynamic corrections obtained from the B3LYP-D3/def2-SVP@CPCM(acetonitrile) level of theory to single point energies obtained at the more accurate B2GP-PLYP-D3/def2-QZVPP@CPCM(acetonitrile) level of theory and using a tightscf convergency criteria. The use of the B2GP-PLYP[8] double hybrid in combination with a large basis set is motivated by the search of as accurate as possible reaction energies and barriers for open-shell transition metal complexes.[9] B2GP-PLYP is a general purpose doubled hybrid functional designed to provide a balanced treatment of both, kinetics and thermochemistry, showing outstanding robustness in several benchmark studies.[10,11] The RIJCOSX[12] approximation has been used to speed-up the Coulomb and exchange parts in the B3LYP calculations, with the def2/J[13] auxiliary basis set. For the double hybrid calculations, the RI-JK approximation was used instead, with the def2/JK[14] auxiliary basis set. The MP2 correlation part was accelerated with the RI-approximation and the def2-QZVPP/C[15] auxiliary basis set. To account for the temperature dependency of the Gibbs free energies, thermochemical corrections have been computed at all the temperatures covered in this work. We made use of the quasi-rigid-rotor harmonic oscillator treatment proposed by Grimme[16] to deal with the description of low energy modes. All these calculations were carried out in the ORCA 5.0.3 software.[17,18]

Suitable guess geometries for the transition state optimizations of the ^{3}MLCT → ^{3}MC reaction pathways were obtained with the nudged elastic band method,[19] as implemented in the pysisyphus[20] software suite version 0.7.5.post1, using ORCA 5.0.3 as the external quantum chemistry code. Hence, the activation barrier of the ^{3}MLCT ⟶ ^{3}MC process is attained by describing it as an adiabatic chemical reaction and not as formally a crossing point between the ^{3}MLCT/ ^{3}MC potential energy surfaces.[21–23] A typical input file with the thresholds used in this work is shown in section S1.1.1. The geometry optimization and hessian calculations of the MECPs were perform from the implemented algorithm in ORCA 5.0.3, which closely resembles that proposed by Harvey et al.[24] This means that the activation barrier for ^{3}MC ⟶ S_0 chemical process is estimated through the crossing point between both potential energy surfaces.[21–23] Orbitals in figure S3 were rendered with IboView v20211019-RevA.[25]

S1.1.1 Nudge elastic band calculations with Pysisyphus

To obtain a suitable guess for the transition state optimizations, nudge elastic band (NEB) calculations were performed with Pysisyphus.[20] The input file settings used in our work are displayed below.

```
geom:
 type: cart
 fn: reactant_product.trj
calc:
 type: orca
 keywords: UKS b3lyp d3zero def2-SVP defgrid2 tightscf slowconv
           normalprint soscf notrah CPCM(acetonitrile)
 blocks: "%scf MaxIter 500 end"
 charge: 2
```

```
 mult: 3
 pal: 64
 mem: 10000
preopt:
 max_cycles: 500
 thresh: gau
 interpol:
 type: redund
 between: 10
cos:
 type: neb
 climb: True
 fix_ends: True
opt:
 type: lbfgs
 align: True
 rms_force: 0.001
 max_step: 0.01
```

These settings proved to be rather robust, as can be seen from Figure S1, where besides smooth convergence, the highest-energy image for ^{3}MC-trans is already very close in relative energy to ^{3}MLCT (8.5 kcal/mol) as the optimized transition stat e(7.5 kcal/mol). Note that we are referring to electronic energies. As for the ^{3}MC-cis and ^{3}MC-twist transition states, the final NEB cycles show a flat potential energy surface around the highest energy image, which explains why it was not possible to converge a geometry optimization to a transition state.

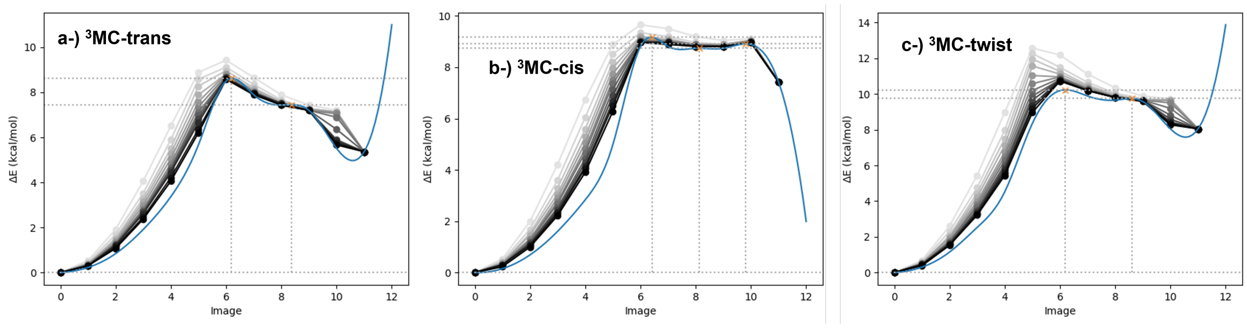

Figure S1: Nudge Elastic Band simulation of the ^{3}MLCT $\rightarrow$ ^{3}MC-trans, ^{3}MLCT $\rightarrow$ ^{3}MC-cis and ^{3}MLCT $\rightarrow$ ^{3}MC-twist pathways.

S1.2 Calculation of the radiative rate

The radiative rate constant $\mathrm{k_r}$ was computed considering the lowest three sub-levels of the ^{3}MLCT state and using a Boltzmann statistics over the levels as:

$$\mathrm{k_r} = \frac{\mathrm{k_1} + \mathrm{k_2} \mathrm{e}^{\frac{-\Delta E_{1,2}}{\mathrm{k_B T}}} + \mathrm{k_3} \mathrm{e}^{\frac{-\Delta E_{1,3}}{\mathrm{k_B T}}}}{1 + \mathrm{e}^{\frac{-\Delta E_{1,2}}{\mathrm{k_B T}}} + \mathrm{e}^{\frac{-\Delta E_{1,3}}{\mathrm{k_B T}}}} \tag{1}$$

There, $\mathrm{k_1}$ is the radiative rate from the first sub-level, $E_{1,2}$ is the energy difference between the first and the second sub-levels, and so on. Each radiative rate k_i for the lowest three-spin sublevels $i(i = 1, 2, 3)$ to the ground state is obtained as (in atomic units),[26]

$$\mathrm{k_i} = \frac{2\mathrm{f_i} \Delta E_{\mathrm{i}}^2}{\mathrm{c}^3} \tag{2}$$

where f is the transition dipole moment and ΔE is the vertical excitation energy dressed with the spin-orbit couplings. Each radiative rate was multiplied by the square of the refractive index of acetonitrile (1.344), following the Strickler–Berg relationship.[27] Radiative rates were obtained within the fully self-consistent spin-orbit coupling time-dependent density functional theory (SOC-TDDFT) treatment[28] of the 4 lowest spin-mixed excitations at the ZORA[29,30]-B3LYP-D4[31,32]/TZP[33]@COSMO[34](acetonitrile) level of theory. These calculations were performed on the optimized geometry of the lowest triplet using the unrestricted

DFT formalism at the ZORA-B3LYP-D4/DZP[33]@COSMO(acetonitrile) level of theory. All the radiative rates calculations were performed using the Amsterdam Density Functional 2020.1 software through the Amsterdam Modeling Suite 2021.1 driver.[35] The chosen settings have been shown to provide an overall good agreement for radiative rates of transition metals, as benchmarked by Mori et al.[36]

S1.3 Calculation of the intersystem crossing rate

The intersystem crossing rate (k_{ISC}) was evaluated using the recently released FCclasses3 software package,[37] suitable for vibronic simulations of electronic spectra and nonradiative rates, based on the harmonic approximation. Specifically, we used the adiabatic Hessian model implemented in FCclasses3.[38] The intersystem crossing rate calculation requires the adiabatic energy difference between the triplet state (^{3}MLCT) and the ground state (S_0), which is 2.05 eV at the B3LYP-D3/def2-SVP@CPCM(acetonitrile) level of theory and the averaged spin–orbit coupling matrix elements between the ^{3}MLCT and the S_0 states (5.33 cm^{-1}).[39] We employed a Gaussian broadening function with a half-width at half-maximum of 0.02 eV.

S2 Further computational results

S2.1 Geometric features of the computed structures

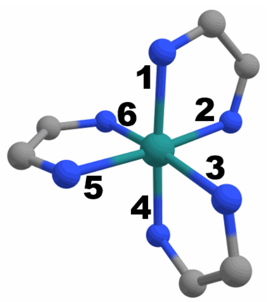

Figure S2: Simplified structure of $[Ru(bpy)_3]^{2+}$ with numbered bond distances (Ru-N) that are reported in Table S1.

Table S1: Relevant bond distances of all molecules computed in this work, following the numeration shown in Figure S2

Bond	Distance (Å)							
	^{3}MLCT	TS (^{3}MC-trans)	^{3}MC-trans	^{3}MC-cis	^{3}MC-twist	^{3}MC/S_0 MECP-trans	^{3}MC/S_0 MECP-cis	^{3}MC/S_0 MECP-twist
1	2.078	2.276	2.451	2.441	2.529	2.504	2.295	2.577
2	2.1	2.135	2.146	2.434	2.193	2.234	2.786	2.151
3	2.099	2.113	2.146	2.084	2.08	2.129	2.074	2.076
4	2.079	2.263	2.452	2.169	2.179	2.825	2.085	2.164
5	2.048	2.081	2.089	2.166	2.316	2.063	2.205	2.339
6	2.049	2.082	2.089	2.084	2.105	2.089	2.079	2.105

We have summarized in table S1 the Ru-N bond distances of the molecular structures computed that describe the non-radiative decay from the emissive ^{3}MLCT through the metal-centered excited states. As it can be seen, the ^{3}MC-trans isomer is characterized by axial elongation of Ru-N bonds that are located in different bipyridyl ligands. This distortion in the ^{3}MC-trans isomer is consistent with the population of a d_{z^2}-like dσ^* orbital (see Figure S3). In the case of the ^{3}MC-cis isomer, the main bond distortion/elongation is at the Ru-N bonds of the same bipyridyl ligand also consistent with population of a $d_{x^2-y^2}$-like dσ^* orbital. This is why population of the ^{3}MC-cis isomer has been deemed relevant for the understanding of ligand photodissociation. The ^{3}MC-twist isomer is characterized by a more complex structural distortion, present over the 3 bipyridyl ligands. This isomer is characterized by population of a $d_{x^2-y^2}$-like dσ^* orbital which explains why its energetic profile is so similar to that of the ^{3}MC-cis isomer. The respective ^{3}MC/S_0 MECP of each isomer have Ru-N bond elongations consistent with that of the respective metal-centered excited states, with the feature that the bond elongation is bigger. Their electronic character from the triplet spin potential energy surface is similar to that of their respective ^{3}MC excited states (see Figure S3). This results are consistent with previous reports on $[Ru(bpy)_3]^{2+}$[22,40,41]

S2.2 Highest energy Single-Occupied Molecular Orbitals.

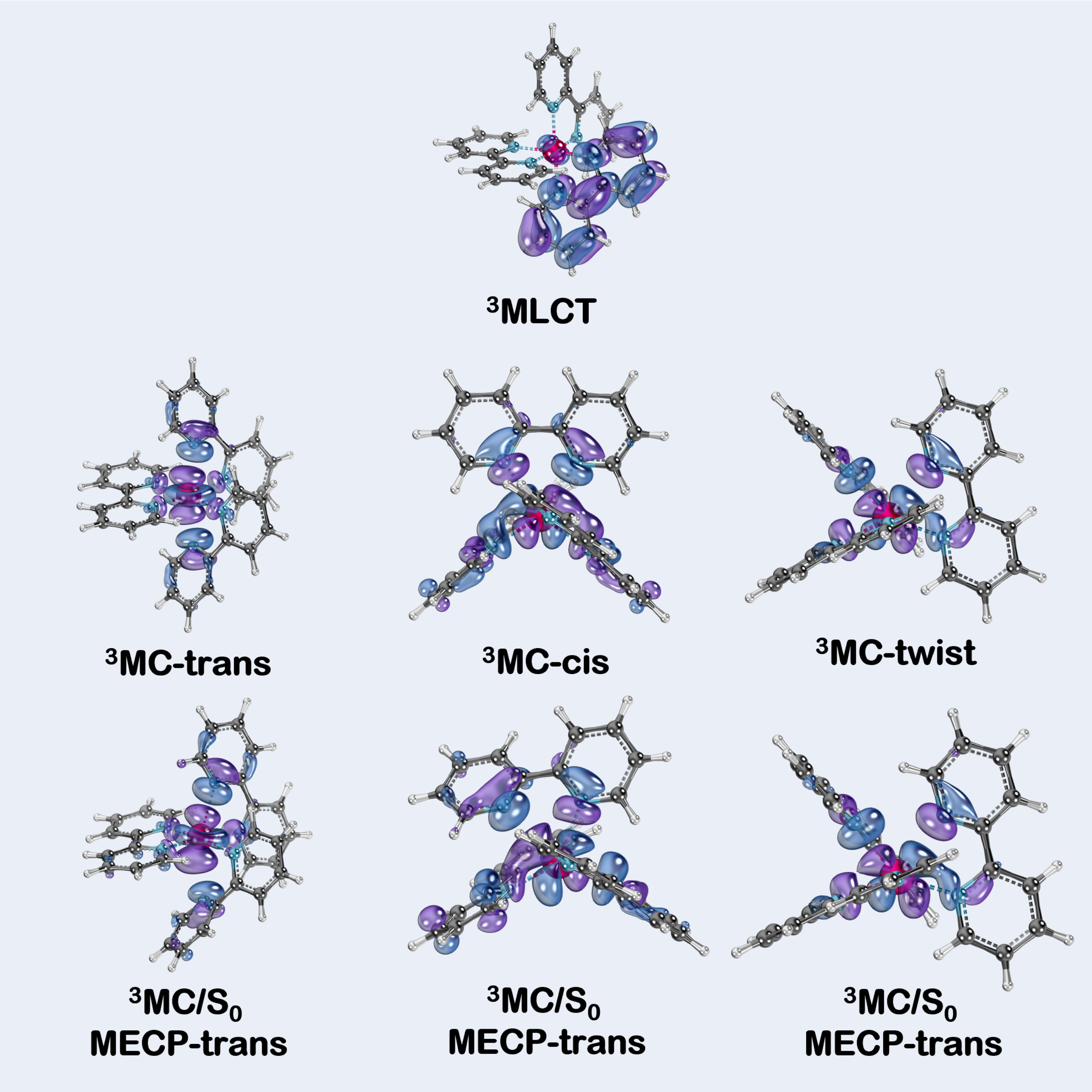

Figure S3: Isosurface plots of the highest energy Singly Occupied Molecular Orbital of relevant structures, as indicated.

S2.3 Simulating the "degree of rate control" with Concvar

Time-dependent concentrations were simulated with the recently published Concvar v1.0 package,[42] which can numerically solve chemical kinetics equations, given the free energy of all minima and transition states involved in an energy profile.

Since through Equation 3 we have access to all rates constants from the computed reaction profiles, we can further perform a simple test of "degree of rate control"[43,44] of any given intermediates and transition states or MECPs.

$$\mathrm{k} = \kappa \frac{\mathrm{k_B T}}{\mathrm{h}} \mathrm{e}^{\frac{-\Delta G^{\neq}}{\mathrm{RT}}} \tag{3}$$

In such a test, we quantify the response of the concentration change of the ^{3}MLCT by doing a differential change of an intermediate or transition state/MECP. If the reaction barrier of the ^{3}MLCT/^{3}MC step controls the rate of the whole decay pathway, then an small change on the $\Delta G^{\neq}_{\mathrm{a}}$ reaction barrier would lead to a change in the time-dependent behaviour of the ^{3}MLCT concentration. Figure S4 illustrates how a 1 kcal/mol increase of the reaction barrier ^{3}MLCT/^{3}MC-trans (while keeping constant the ^{3}MLCT/^{3}MC-trans equilibrium constant and the ^{3}MC-trans//^{3}MC/S_0 MECP energy difference) does not change the consumption rate of the ^{3}MLCT (green curve). However, the same 1 kcal/mol increase for the ^{3}MC-trans//^{3}MC/S_0 MECP energy difference does alters the consumption rate of the ^{3}MLCT (blue curve).

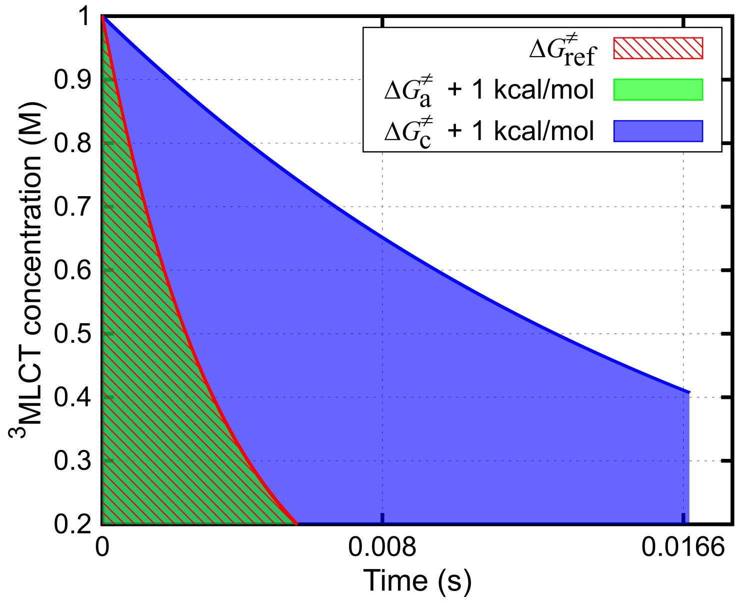

Figure S4: Concentration variation of the ^{3}MLCT, as simulated with Concvar. In dash red are the results from the computed energy profile for the ^{3}MC-trans JT isomer, which we take as a reference, in green the results for a 1 kcal/mol increase in the Gibbs free energy reaction barrier of the TS-trans and in blue for a 1 kcal/mol increase in the Gibbs free energy ^{3}MC/^{1}S$_0$ MECP-trans.

Explanations on the keywords used by Concvar to solve numerically the chemical kinetics equations and obtain time-dependent concentrations can be found in its user manual.

S2.4 Temperature-dependent emission lifetimes.

As discussed in the manuscript, in $[Ru(bpy)_3]^{2+}$, the dominant view in the literature is to consider the ^{3}MC-trans Jahn-Teller isomer for explaining the luminescence lifetimes of the ^{3}MLCT excited state. Additionally, the energy gap or energy barrier between the ^{3}MLCT $\longrightarrow$ ^{3}MC excited states have been established as the main quantities required to rationalize emission lifetimes. For comparison, here we plot (Figure S5) what would be the theoretical prediction of emission lifetimes if instead of using k_{nr} (^{3}MLCT $\longrightarrow$ ^{3}MC $\longrightarrow$ S_0) one uses k_a (^{3}MLCT $\longrightarrow$ ^{3}MC; recall equations 3 and 5 in the manuscript). Lifetimes shown in Figure S5-a are obtained using experimental values of k_r and k_{ISC} and theoretical values of either k_{nr} (solid lines) or k_a (discontinuous lines). Lifetimes shown in Figure S5-b are

obtained using using solely theoretically derived values for all rates, which means k_r and k_{ISC} incorporate now a temperature dependency.

Lifetimes predicted when considering a non-radiative decay through the prototypical ^{3}MC-trans and with a rate constant quantified as k_a (discontinuous blue line in Figure S5-a and -b) are not visible since they overlap with the results obtained when considering the decay through all 3 isomers, each quantified by its respective k_a rate (discontinuous red line in Figure S5-a and -b). The considerable drop of the lifetime when considering only the ^{3}MC-trans isomer (in either panel -a or -b of Figure S5) is not surprising since k_a is related to the reaction barrier ^{3}MLCT $\longrightarrow$ ^{3}MC step, which has an associated reaction barrier of only 7.2 kcal/mol. Lifetimes predicted when considering only either the ^{3}MC-cis or ^{3}MC-twist are similar to those from predicted from their k_{nr} since the crossing points to the ground state are almost iso-energetic to the respective MC excited states. More importantly, when all three Jahn-Teller isomers are considered (-trans, -cis and -twist) the predicted lifetime is almost zero (discontinuous red lines) because k_a from the ^{3}MC-trans isomer would considerably dominate over that of the -cis and -twist isomers.

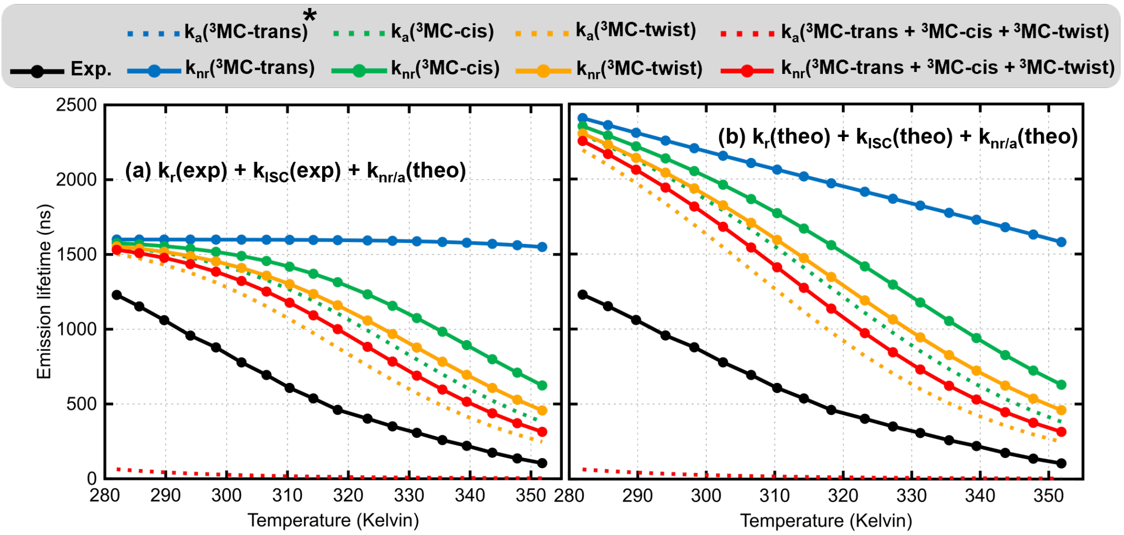

Figure S5: Temperature-dependent emission lifetimes of $[Ru(bpy)_3]^{2+}$ in ns. Experimental values are given by the black line. Blue, green and orange are the computational lifetimes obtained using the non-radiative rates k_{nr} (solid lines) or k_a (discontinuous lines) of the ^{3}MC-trans, ^{3}MC-cis or ^{3}MC-twist isomers, respectively. Red solid line considers all three pathways, as in equation 6 of the manuscript (discontinuous lines for k_a). Panel (a) shows computational results obtained using experimental k_r and k_{ISC} values. Panel (b) uses theoretically calculated k_r and k_{ISC} values. *Lifetimes when considering only the decay through the ^{3}MC-trans and with a rate quantified by k_a (blue discontinuous line), are not visible because they overlap with the discontinuous red line.

S2.5 Computed Gibbs free energies for the ^{3}MC-trans, ^{3}MC-cis and ^{3}MC-twist isomers, over a range of temperatures.

Despite the fact that most studies computing the decay through the ^{3}MC states are build upon the use of hybrid functionals, Zhang et al.[21] have shown how such functionals are prone to underestimate reaction barriers. We have computed the reaction barriers for each decay pathway (through the $^3\text{MC}-\text{trans}$, $^3\text{MC}-\text{cis}$ and $^3\text{MC}-\text{twist}$ JT isomers) at the B3LYP-D3/def2-QZVPP@CPCM(acetonitrile)//B3LYP-D3/def2-SVP@CPCM(acetonitrile) (Tables S2, S3 and S4) and at the B2GP-PLYP-D3/def2-QZVPP@CPCM(acetonitrile)//B3LYP-D3/def2-SVP@CPCM(acetonitrile) (Tables S5, S6 and S7) level of theory.

As it can be seen, at the B3LYP-D3/def2-QZVPP@CPCM(acetonitrile)//B3LYP-D3/def2-SVP@CPCM(acetonitrile) level of theory, the energetic span for each decay is severely un-

derestimated and it would be impossible to get even a semi-quantitative agreement with the experimental lifetimes. Indeed the use of the double-hybrid functional rises the relative energies while keeping the qualitative behavior between each decay pathway the same.

Table S2: Relative energies with respect to the (^{3}MLCT) state (0.0 kcal/mol) for the decay through the ^{3}MC-trans at different temperatures, at the B3LYP-D3/def2-QZVPP@CPCM(acetonitrile)//B3LYP-D3/def2-SVP@CPCM(acetonitrile) level of theory.

Temp.(K)	$\Delta\Delta G(\mathrm{TS-trans})$	$\Delta\Delta G(^3\mathrm{MC-trans})$	$\Delta\Delta G$ ^{3}MC/^{1}S$_0$ MECP
281.95	6.47	5.21	11.07
285.65	6.47	5.20	11.05
289.75	6.46	5.18	11.02
294.05	6.45	5.16	11.00
298.25	6.45	5.14	10.98
302.45	6.44	5.13	10.95
306.55	6.44	5.11	10.93
310.35	6.43	5.09	10.91
314.25	6.42	5.08	10.89
318.25	6.42	5.06	10.87
323.15	6.41	5.04	10.84
327.25	6.40	5.02	10.82
331.25	6.40	5.01	10.80
335.45	6.39	4.99	10.78
339.45	6.39	4.97	10.75
343.65	6.38	4.96	10.73
347.75	6.38	4.94	10.71
351.85	6.37	4.92	10.69

Table S3: Relative energies respect to the (^{3}MLCT) state (0.0 kcal/mol) for the decay through the ^{3}MC-cis, at different temperatures, at the B3LYP-D3/def2-QZVPP@CPCM(acetonitrile)//B3LYP-D3/def2-SVP@CPCM(acetonitrile) level of theory.

Temp.(K)	$\Delta\Delta G(\mathrm{TS-cis})$	$\Delta\Delta G(^3\mathrm{MC-cis})$	$\Delta\Delta G$ ^{3}MC/^{1}S$_0$ MECP
281.95	7.78	7.45	8.28
285.65	7.78	7.43	8.26
289.75	7.77	7.40	8.24
294.05	7.77	7.38	8.22
298.25	7.76	7.36	8.19
302.45	7.76	7.33	8.17
306.55	7.76	7.31	8.15
310.35	7.75	7.29	8.13
314.25	7.75	7.27	8.11
318.25	7.75	7.25	8.09
323.15	7.74	7.22	8.06
327.25	7.74	7.20	8.04
331.25	7.73	7.18	8.02
335.45	7.73	7.16	8.00
339.45	7.73	7.14	7.98
343.65	7.73	7.12	7.95
347.75	7.72	7.10	7.93
351.85	7.72	7.07	7.91

Table S4: Relative energies with respect to the (^{3}MLCT) state (0.0 kcal/mol) for the decay through the ^{3}MC-twist, at different temperatures, at the B3LYP-D3/def2-QZVPP@CPCM(acetonitrile)//B3LYP-D3/def2-SVP@CPCM(acetonitrile) level of theory.

Temp.(K)	$\Delta\Delta G$(TS − twist)	$\Delta\Delta G$(^{3}MC − twist)	$\Delta\Delta G$ ^{3}MC/^{1}S$_0$ MECP
281.95	8.27	7.95	8.31
285.65	8.25	7.93	8.29
289.75	8.24	7.91	8.27
294.05	8.23	7.89	8.25
298.25	8.22	7.87	8.24
302.45	8.21	7.85	8.22
306.55	8.20	7.83	8.20
310.35	8.19	7.81	8.18
314.25	8.18	7.79	8.17
318.25	8.16	7.77	8.15
323.15	8.15	7.75	8.13
327.25	8.14	7.73	8.11
331.25	8.13	7.71	8.09
335.45	8.12	7.69	8.07
339.45	8.11	7.67	8.06
343.65	8.10	7.65	8.04
347.75	8.09	7.63	8.02
351.85	8.08	7.62	8.00

Table S5: Relative energies with respect to the (^{3}MLCT) state (0.0 kcal/mol) for the decay through the ^{3}MC-trans, at different temperatures, at the B2GP-PLYP-D3/def2-QZVPP@CPCM(acetonitrile)//B3LYP-D3/def2-SVP@CPCM(acetonitrile) level of theory.

Temp.(K)	$\Delta\Delta G$(TS − trans)	$\Delta\Delta G$(^{3}MC − trans)	$\Delta\Delta G$ ^{3}MC/^{1}S$_0$ MECP
281.95	7.21	6.98	14.16
285.65	7.21	6.96	14.14
289.75	7.20	6.95	14.11
294.05	7.19	6.93	14.09
298.25	7.19	6.91	14.07
302.45	7.18	6.89	14.04
306.55	7.17	6.87	14.02
310.35	7.17	6.86	14.00
314.25	7.16	6.84	13.98
318.25	7.16	6.83	13.96
323.15	7.15	6.80	13.93
327.25	7.14	6.79	13.91
331.25	7.14	6.77	13.89
335.45	7.13	6.75	13.87
339.45	7.13	6.74	13.85
343.65	7.12	6.72	13.82
347.75	7.12	6.70	13.80
351.85	7.11	6.69	13.78

Table S6: Relative energies with respect to the (^{3}MLCT) state (0.0 kcal/mol) for the decay through the ^{3}MC-cis, at different temperatures, at the B2GP-PLYP-D3/def2-QZVPP@CPCM(acetonitrile)//B3LYP-D3/def2-SVP@CPCM(acetonitrile) level of theory.

Temp.(K)	$\Delta\Delta G(^3\mathrm{MC-cis})$	$\Delta\Delta G$ ^{3}MC/^{1}S$_0$ MECP
281.95	10.94	10.97
285.65	10.92	10.95
289.75	10.89	10.93
294.05	10.87	10.90
298.25	10.85	10.88
302.45	10.82	10.86
306.55	10.80	10.84
310.35	10.78	10.82
314.25	10.76	10.80
318.25	10.74	10.78
323.15	10.71	10.75
327.25	10.69	10.73
331.25	10.67	10.71
335.45	10.65	10.69
339.45	10.63	10.67
343.65	10.61	10.64
347.75	10.59	10.62
351.85	10.57	10.60

Table S7: Relative energies with respect to the (^{3}MLCT) state (0.0 kcal/mol) for the decay through the ^{3}MC-twist, at different temperatures, at the B2GP-PLYP-D3/def2-QZVPP@CPCM(acetonitrile)//B3LYP-D3/def2-SVP@CPCM(acetonitrile) level of theory.

Temp.(K)	$\Delta\Delta G(^3\mathrm{MC-twist})$	$\Delta\Delta G$ ^{3}MC/^{1}S$_0$ MECP
281.95	10.53	10.62
285.65	10.51	10.60
289.75	10.49	10.58
294.05	10.47	10.56
298.25	10.45	10.54
302.45	10.43	10.53
306.55	10.41	10.51
310.35	10.39	10.49
314.25	10.37	10.47
318.25	10.35	10.46
323.15	10.33	10.43
327.25	10.31	10.42
331.25	10.29	10.40
335.45	10.27	10.38
339.45	10.25	10.36
343.65	10.23	10.35
347.75	10.21	10.33
351.85	10.19	10.31

S2.6 Computed non-radiative (k_{nr}) rates.

Table S8: Computed non-radiative rates for the decay through the ^{3}MC-trans, ^{3}MC-cis and ^{3}MC-twist states at different temperatures, at the B2GP-PLYP-D3/def2-QZVPP@CPCM(acetonitrile)//B3LYP-D3/def2-SVP@CPCM(acetonitrile) level of theory.

Temp.(K)	$k_{nr}(^3MC - trans)(s^{-1})$	$k_{nr}(^3MC - cis)(s^{-1})$	$k_{nr}(^3MC - twist)(s^{-1})$
281.95	62	950	1.86E+04
285.65	91	12842	24837
289.75	136	17782	33922
294.05	206	24800	46608
298.25	306	33989	63004
302.45	448	46137	84434
306.55	644	61739	111493
310.35	894	80260	143371
314.25	1242	104453	184308
318.25	1724	135855	237045
323.15	2548	185926	319926
327.25	3499	239762	407981
331.25	4742	305709	514852
335.45	6463	392097	652887
339.45	8617	493816	813898
343.65	11588	626117	1021359
347.75	15354	784680	1267399
351.85	20194	977923	1564272

S2.7 Computed radiative (k_r) and intersystem-crossing (k_{ISC}) rates

Computed values of k_r and k_{ISC} are shown in Table S9.

Table S9: Computed radiative (k_r) and intersystem-crossing (k_{ISC}) rates from the ^{3}MLCT state to the ground state, at different temperatures.

Temp.(K)	$k_r(s^{-1})$	$k_{ISC}(s^{-1})$
281.95	1.7487E+05	2.407E+05
285.65	1.7590E+05	2.479E+05
289.75	1.7702E+05	2.561E+05
294.05	1.7816E+05	2.649E+05
298.25	1.7925E+05	2.739E+05
302.45	1.8031E+05	2.833E+05
306.55	1.8133E+05	2.927E+05
310.35	1.8225E+05	3.017E+05
314.25	1.8317E+05	3.113E+05
318.25	1.8410E+05	3.214E+05
323.15	1.8521E+05	3.344E+05
327.25	1.8611E+05	3.456E+05
331.25	1.8698E+05	3.569E+05
335.45	1.8787E+05	3.692E+05
339.45	1.8870E+05	3.813E+05
343.65	1.8955E+05	3.945E+05
347.75	1.9036E+05	4.078E+05
351.85	1.9116E+05	4.216E+05

An interesting advantage provided by our calculations is that further insight can be gained regarding the origin of k_{ISC}. Besides the spin-orbit coupling and the adiabatic energy difference between the ^{3}MLCT and the S_0 states, the reorganization energy from the ^{3}MLCT $\rightarrow S_0$ transition has a dominant weight on the magnitude of k_{ISC}. The reorganization energy λ_k of the kth normal mode is related to its Huang–Rhys factor[45] (HR_k) through $\lambda_k = \hbar\omega_k HR_k$.[46] By decomposing the reorganization energy across all normal modes, one can gain insights into which of these mode are strongly coupled between the initial (^{3}MLCT) and final state S_0, which can be exploited for the rational design of more efficient emitters. Caspar and Meyer estimated the main vibrational mode dominating the decay channel to be between 1300 - 1400 cm^{-1}.[47] Our theoretical reorganization energies projected over the

normal modes of the ^{3}MLCT are shown in Figure S6. As it can be seen, there is a main normal mode contributing to k_{ISC} located at 1556 cm^{-1}. Since density functionals tend to slightly overestimate vibrational frequencies, considering an scale factor of 0.967 for the used level of theory,[48] the scaled vibration would lie at about 1504 cm^{-1}, which is close to the estimated experimental values range. It should be noted that experimental values depend on many assumptions,[47] which can have unforeseen impacts on these estimations. Another interesting result from our calculations is the presence of two more normal modes with a sizable contribution to the crossing. All three modes correspond to mainly aromatic C=C stretching and in-plane deformations of a bpy ligand. Following these methodology, further studies could be carry out to discern the influence of different substituents on the bpy ligand to provided general structure/properties relationships for the fine-tuning of k_{ISC}.

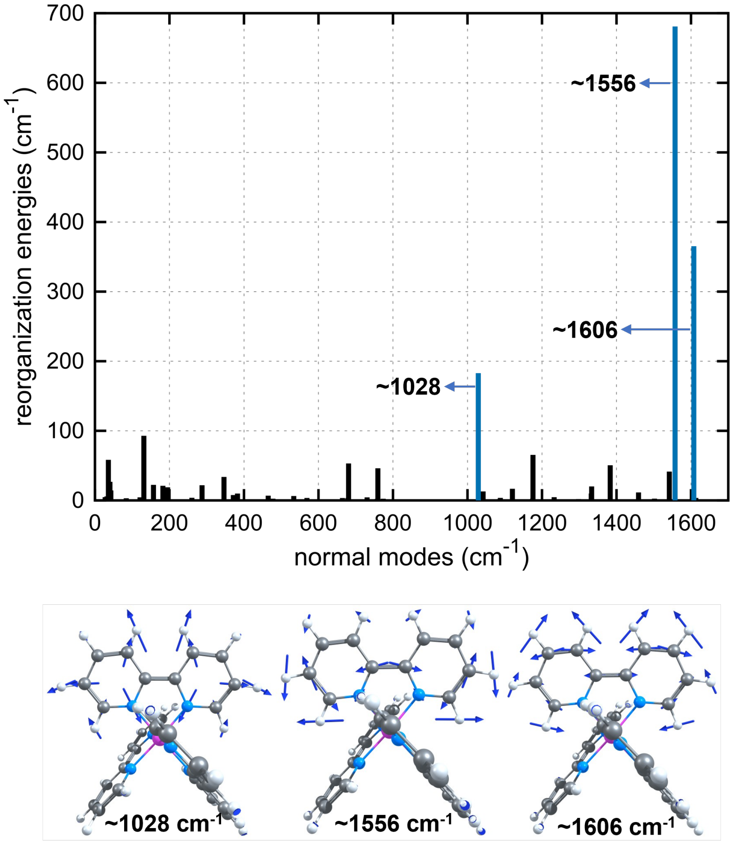

Figure S6: On the top, computed reorganization energies projected over the normal modes of the ^{3}MLCT of $[Ru(bpy)_3]^{2+}$ and on the bottom, displacement vectors of the three dominant vibrations.

S3 Experimental Details

S3.1 Synthesis of $[Ru(bpy)_3](PF_6)_2$

To a solution of 25 mg of $RuCl_3$ (0.12 mmol, 1 eq.) in 20 ml ethanol and 7 ml water the bpy ligand (0.36 mmol, 3 eq.) were added. The mixture was heated in the microwave 2 min at 250 W and 2 h at 200 W. The ethanol was removed by evaporation. After adding 390 mg NH_4PF_6 (2.4 mmol, 20 eq.) dissolved in 5 ml water, the complex was filtered off. The crude product was purified by SEPHADEX column size exclusion chromatography (LH-20 solid phase, methanol liquid phase, 3.5 cm diameter and 75 cm length). The main fraction was purified by slow diffusion of diethyl ether into a acetonitrile solution to yield a red solid. To ensure high purity we treated the yield incidental.
Yield 13% (0.016 mmol, 13.12 mg)

S3.2 NMR spectroscopy

NMR spectra were recorded on a Bruker Avance 400 MHz, or a Bruker Avance 500 MHz, at 298 K and processed with MestreNova software (version 12.0.0). Chemical shifts δ are reported in parts per million (ppm). The ^{1}H- and ^{13}C-NMR shifts are referenced using the deuterated solvent as internal standard. Coupling constants J are presented as absolute values in Hz. For the characterization of the NMR signals the following abbreviations are used: s = singlet, d = doublet, m = multiplet and dd = doublet of doublet, ddd = doublet of doublet of doublet, td = triplet of doublet, hept = heptet.

^{1}**H-NMR** (400 MHz, ACN [D3], 25°C, TMS): δ [ppm] = 8.51 (dd, J = 8.1, 0.8 Hz, 6H), 8.06 (td, J = 8.1, 1.5 Hz, 6H), 7.74 (ddd, J = 5.6, 1.4, 0.7 Hz, 6H), 7.40 (ddd, J = 7.6, 5.6, 1.3 Hz, 6H).
^{13}**C-NMR** (101 MHz, ACN [D3], 25°C, TMS): δ [ppm] = 157.92 (s), 152.61 (s), 138.72 (s),

128.51 (s), 125.19 (s).

^{31}**P-NMR** (162 MHz, ACN [D3], 25°C, TMS): δ [ppm] = -144.47 (hept, J = 706.8 Hz).

S3.3 Time-resolved spectroscopy

Temperature dependent time-resolved emission spectroscopy was measured using a Q-switched pulsed Nd:YAG laser (Q-smart 450mJ, Quantel laser) and VWR Collection MRX-07 Thermostate. All laser pulses had a pulse duration of approx. 6 ns (repetition rate of 10 Hz), and excitation pulses of the Nd:YAG output were centered at 355 nm (laser line filter CWL = 355 ± 2 nm, FWHM = 10 ± 2 nm) to ensure and the power of the pump beam was approximately 1.1 mJ per pulse at the sample. All emission lifetimes were recorded in inert acetonitrile solutions, in a 1 cm quartz cell cuvette. The solvent was of spectroscopic grade. The sample had an optical density of 0.04 at 355 nm and 0.1 at 450 nm. All spectra were recorded at the emission maximum of the sample at 606 nm. As detector a photo multiplier tube of the LP980 spectrometer from Edinburgh Instruments was applied. The temperature in the thermostat bath was changed in 5 °C steps from 5 °C to 90 °C. Every temperature was kept for five minutes before the lifetime measurement. The temperature in the cuvette was measured in a calibration experiment beforehand (see Table S10). All shown temperatures are referenced as the temperature in the cuvette. The temperature stability of the sample was checked by UV/vis measurements before and after the temperature dependent emission lifetime measured with a JASCO V-770 spectrometer and by cooling down the sample and reproducing emission lifetime measurements.

Table S10: Temperature calibration experiment for ACN in the lifetime measured set up.

Thermostat [°C]	Cuvette [°C]
5	8.8
10	12.2
15	16.2
20	20.5
25	25
30	29.1
35	33.2
40	37.1
45	41
50	45.1
55	49.3
60	53.9
65	58
70	62.3
75	66.3
80	70.4
85	74.6
90	78.7

Steady-state absorption and emission spectra were recorded with a JASCO V-670 spectrophotometer and a JASCO FP-8500 spectrofluorometer. All sample were measured under inert conditions in cuvettes with a pathlength of 10 mm.

Photostability tests were carried out with a homebuilt blue LED-stick (as described in a recent work)[49] to irradiate the sample with 465 nm light. The samples were heated in heating bath, while being irradiated from below with a minimum distance to the light source. The measurements were carried out under inert condition. The sample were prepared in cuvettes with a pathlength of 10 mm were used.

Table S11: Maximums peaks for the absorption and emission spectra

	$\lambda_{abs}(nm)$	λ_{em} (nm)
$[Ru(bpy)_3]^{2+}$	286, 321 (sh), 353 (sh), 390 (sh), 420 (sh), 450	606

S3.4 Emission lifetime data and statistic data fit

All emission lifetime data was fitted with R-Studio Version 1.3.959 with a mono-exponential fit:

$$I(x) = a_0 + a_1 * e^{\frac{-x}{\tau_1}} \tag{4}$$

All spectra of a temperature dependent measurement were fitted with the same starting parameters.

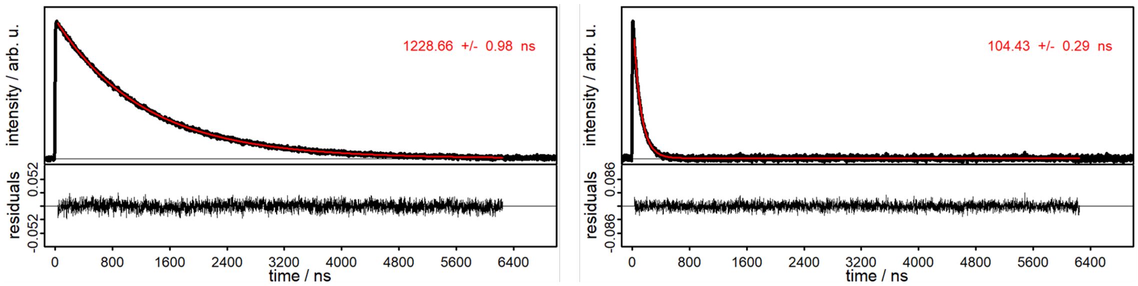

Figure S7: Exponential lifetime fit on $[Ru(bpy)_3]^{2+}$ in inert acetonitrile by 8.8 °C (left) and 78.7 °C (right) with fitting residuals.

Table S12: Emission lifetimes values as obtained by the fits perform in the studied temperature range.

Temp (°C)	Temp (K)	Lifetime (ns)	Variation (ns)
8.8	282.0	1228.7	0.98
12.5	285.7	1150.8	0.96
16.6	289.8	1059.6	0.90
20.9	294.1	956.4	0.83
25.1	298.3	877.4	0.76
29.3	302.5	777.4	0.67
33.4	306.6	693.4	0.64
37.2	310.4	607.2	0.60
41.1	314.3	537.1	0.54
45.1	318.3	460.2	0.48
50.0	323.2	401.5	0.43
54.1	327.3	349.7	0.39
58.1	331.3	307.1	0.41
62.3	335.5	258.4	0.39
66.3	339.5	220.0	0.37
70.5	343.7	174.3	0.35
74.6	347.8	136.2	0.33
78.7	351.9	104.4	0.29
66.3	339.5	185.4	0.35
45.1	318.3	360.3	0.43
25.1	298.3	744.3	0.65

S3.5 Arrhenius-like fit of temperature dependent lifetimes

Having at hand the lifetimes of $[Ru(bpy)_3]^{2+}$ in a wide range of temperatures, we can proceed to perform an Arrhenius-like fit.[47] To this end, we make use of the following equation:

$$\tau_{\text{emission}}(\mathrm{T}) = \frac{1}{\mathrm{k} + \mathrm{A}e^{\frac{-\Delta E}{\mathrm{RT}}}} \tag{5}$$

Through this fit, one can obtain values for the pre-exponential value A, ΔE and k ($k = k_r + k_{ISC}$). The results of such fit are provided in Figure S8.

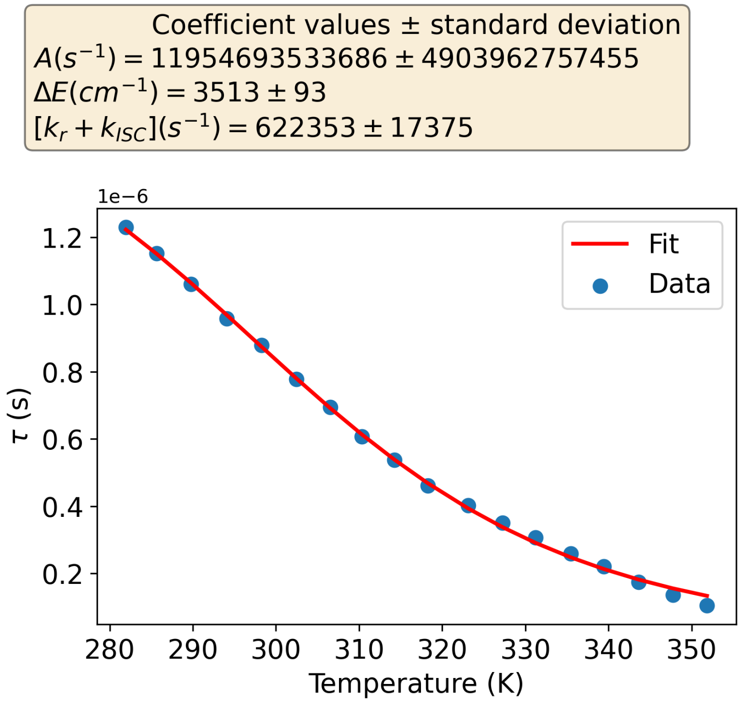

Figure S8: Fitting of the excited-state lifetime with temperature following equation 5

Note that to properly determine the individual values of $\mathrm{k_r}$ and $\mathrm{k_{ISC}}$, one additionally needs to perform temperature-dependent quantum yield experiments and make additional use of the equation:

$$\Phi(T) = \frac{k_r}{k_r + k_{ISC} + k_{nr}(T)} \tag{6}$$

where k_{nr} is:

$$\mathrm{k_{nr}} = \mathrm{A}\mathrm{e}^{\frac{-\Delta E}{\mathrm{RT}}} \tag{7}$$

However, for the purposes of this work, independent determination of $\mathrm{k_r}$ and $\mathrm{k_{ISC}}$ are not necessary. We were only interested in their added values (as obtained through the fit of equation 5) to be combined with the theoretical values of $\mathrm{k_{nr}}$, which altogether provides the results shown in Figure 5a of the manuscript. More detailed analysis will be performed in

due course, taking temperature dependent quantum yield determinations into account.

References

(1) Becke, A. D. Density-functional thermochemistry. III. The role of exact exchange. *J. Chem. Phys.* **1993**, *98*, 5648–5652.

(2) Lee, C.; Yang, W.; Parr, R. G. Development of the Colle-Salvetti correlation-energy formula into a functional of the electron density. *Phys. Rev. B* **1988**, *37*, 785–789.

(3) Weigend, F.; Ahlrichs, R. Balanced basis sets of split valence, triple zeta valence and quadruple zeta valence quality for H to Rn: Design and assessment of accuracy. *Phys. Chem. Chem. Phys.* **2005**, *7*, 3297–3305.

(4) Peterson, K. A.; Figgen, D.; Dolg, M.; Stoll, H. Energy-consistent relativistic pseudopotentials and correlation consistent basis sets for the 4d elements Y-Pd. *J. Chem. Phys.* **2007**, *126*, 124101.

(5) Grimme, S.; Antony, J.; Ehrlich, S.; Krieg, H. A consistent and accurate ab initio parametrization of density functional dispersion correction (DFT-D) for the 94 elements H-Pu. *J. Chem. Phys.* **2010**, *132*, 154104.

(6) Barone, V.; Cossi, M. Quantum Calculation of Molecular Energies and Energy Gradients in Solution by a Conductor Solvent Model. *J. Phys. Chem. A* **1998**, *102*, 1995–2001.

(7) Cossi, M.; Rega, N.; Scalmani, G.; Barone, V. Energies, structures, and electronic properties of molecules in solution with the C-PCM solvation model. *J. Comput. Chem.* **2003**, *24*, 669–681.

(8) Karton, A.; Tarnopolsky, A.; Lamère, J. F.; Schatz, G. C.; Martin, J. M. Highly accurate first-principles benchmark data sets for the parametrization and validation of density functional and other approximate methods. Derivation of a robust, generally applicable, double-hybrid functional for thermochemistry and thermochemical . *J. Phys. Chem. A*

2008, *112*, 12868–12886.

(9) Maurer, L. R.; Bursch, M.; Grimme, S.; Hansen, A. Assessing Density Functional Theory for Chemically Relevant Open-Shell Transition Metal Reactions. *J. Chem. Theory Comput.* **2021**, *17*, 6134–6151.

(10) Goerigk, L.; Hansen, A.; Bauer, C.; Ehrlich, S.; Najibi, A.; Grimme, S. A look at the density functional theory zoo with the advanced GMTKN55 database for general main group thermochemistry, kinetics and noncovalent interactions. *Phys. Chem. Chem. Phys.* **2017**, *19*, 32184–32215.

(11) Mehta, N.; Casanova-Páez, M.; Goerigk, L. Semi-empirical or non-empirical double-hybrid density functionals: Which are more robust? *Phys. Chem. Chem. Phys.* **2018**, *20*, 23175–23194.

(12) Neese, F.; Wennmohs, F.; Hansen, A.; Becker, U. Efficient, approximate and parallel Hartree-Fock and hybrid DFT calculations. A 'chain-of-spheres' algorithm for the Hartree-Fock exchange. *Chem. Phys.* **2009**, *356*, 98–109.

(13) Weigend, F. Accurate Coulomb-fitting basis sets for H to Rn. *Phys. Chem. Chem. Phys.* **2006**, *8*, 1057–1065.

(14) Weigend, F. Hartree-fock exchange fitting basis sets for H to Rn. *J. Comput. Chem.* **2008**, *29*, 167–175.

(15) Hellweg, A.; Hättig, C.; Höfener, S.; Klopper, W. Optimized accurate auxiliary basis sets for RI-MP2 and RI-CC2 calculations for the atoms Rb to Rn. *Theor. Chem. Acc.* **2007**, *117*, 587–597.

(16) Grimme, S. Supramolecular binding thermodynamics by dispersion-corrected density functional theory. *Chem. - A Eur. J.* **2012**, *18*, 9955–9964.

(17) Neese, F.; Wennmohs, F.; Becker, U.; Riplinger, C. The ORCA quantum chemistry program package. *J. Chem. Phys.* **2020**, *152*, 224108.

(18) Neese, F. Software update: The ORCA program system—Version 5.0. *Wiley Interdiscip. Rev. Comput. Mol. Sci.* **2022**, *12*, e1606.

(19) Jónsson, H.; Mills, G.; Jacobsen, K. W. Nudged elastic band method for finding minimum energy paths of transitions. World Sci. Singapore. 1998; pp 385–404.

(20) Steinmetzer, J.; Kupfer, S.; Gräfe, S. pysisyphus: Exploring potential energy surfaces in ground and excited states. *Int. J. Quantum Chem.* **2021**, *121*, e26390.

(21) Zhang, X.; Jacquemin, D.; Peng, Q.; Shuai, Z.; Escudero, D. General Approach to Compute Phosphorescent OLED Efficiency. *J. Phys. Chem. C* **2018**, *122*, 6340–6347.

(22) Soupart, A.; Dixon, I. M.; Alary, F.; Heully, J. L. DFT rationalization of the room-temperature luminescence properties of Ru(bpy) 32+ and Ru(tpy) 22+ : 3MLCT–3MC minimum energy path from NEB calculations and emission spectra from VRES calculations. *Theor. Chem. Acc.* **2018**, *137*, 37.

(23) Soupart, A.; Alary, F.; Heully, J. L.; Elliott, P. I.; Dixon, I. M. Recent progress in ligand photorelease reaction mechanisms: Theoretical insights focusing on Ru(II) 3MC states. *Coord. Chem. Rev.* **2020**, *408*, 213184.

(24) Harvey, J. N.; Aschi, M.; Schwarz, H.; Koch, W. The singlet and triplet states of phenyl cation. A hybrid approach for locating minimum energy crossing points between non-interacting potential energy surfaces. *Theor. Chem. Acc.* **1998**, *99*, 95–99.

(25) Knizia, G.; Klein, J. E. Electron flow in reaction mechanisms - Revealed from first principles. *Angew. Chemie - Int. Ed.* **2015**, *54*, 5518–5522.

(26) Baryshnikov, G.; Minaev, B.; Ågren, H. Theory and Calculation of the Phosphorescence Phenomenon. *Chem. Rev.* **2017**, *117*, 6500–6537.

(27) Strickler, S. J.; Berg, R. A. Relationship between absorption intensity and fluorescence lifetime of molecules. *J. Chem. Phys.* **1962**, *37*, 814–822.

(28) Wang, F.; Ziegler, T.; Van Lenthe, E.; Van Gisbergen, S.; Baerends, E. J. The calculation of excitation energies based on the relativistic two-component zeroth-order regular approximation and time-dependent density-functional with full use of symmetry. *J. Chem. Phys.* **2005**, *122*, 204103.

(29) Van Lenthe, E.; Baerends, E. J.; Snijders, J. G. Relativistic regular two-component

Hamiltonians. *J. Chem. Phys.* **1993**, *99*, 4597–4610.

(30) Van Lenthe, E.; Baerends, E. J.; Snijders, J. G. Relativistic total energy using regular approximations. *J. Chem. Phys.* **1994**, *101*, 9783–9792.

(31) Caldeweyher, E.; Bannwarth, C.; Grimme, S. Extension of the D3 dispersion coefficient model. *J. Chem. Phys.* **2017**, *147*, 034112.

(32) Caldeweyher, E.; Ehlert, S.; Hansen, A.; Neugebauer, H.; Spicher, S.; Bannwarth, C.; Grimme, S. A generally applicable atomic-charge dependent London dispersion correction. *J. Chem. Phys.* **2019**, *150*, 154122.

(33) Van Lenthe, E.; Baerends, E. J. Optimized Slater-type basis sets for the elements 1-118. *J. Comput. Chem.* **2003**, *24*, 1142–1156.

(34) Pye, C. C.; Ziegler, T. An implementation of the conductor-like screening model of solvation within the Amsterdam density functional package. *Theor. Chem. Acc.* **1999**, *101*, 396–408.

(35) te Velde, G.; Bickelhaupt, F. M.; Baerends, E. J.; Fonseca Guerra, C.; van Gisbergen, S. J.; Snijders, J. G.; Ziegler, T. Chemistry with ADF. *J. Comput. Chem.* **2001**, *22*, 931–967.

(36) Mori, K.; Goumans, T. P.; Van Lenthe, E.; Wang, F. Predicting phosphorescent lifetimes and zero-field splitting of organometallic complexes with time-dependent density functional theory including spin-orbit coupling. *Phys. Chem. Chem. Phys.* **2014**, *16*, 14523–14530.

(37) Cerezo, J.; Santoro, F. FCclasses3: Vibrationally-resolved spectra simulated at the edge of the harmonic approximation. *J. Comput. Chem.* **2023**, *44*, 626–643.

(38) Avila Ferrer, F. J.; Santoro, F. Comparison of vertical and adiabatic harmonic approaches for the calculation of the vibrational structure of electronic spectra. *Phys. Chem. Chem. Phys.* **2012**, *14*, 13549–13563.

(39) Guo, H.; Dang, C.; Zhao, J.; Dick, B. Lighting the flavin decorated ruthenium(II) polyimine complexes: A theoretical investigation. *Inorg. Chem.* **2019**, *58*, 8486–8493.

(40) Soupart, A.; Alary, F.; Heully, J. L.; Elliott, P. I.; Dixon, I. M. Exploration of Uncharted 3PES Territory for [Ru(bpy)3]2+: A New 3MC Minimum Prone to Ligand Loss Photochemistry. *Inorg. Chem.* **2018**, *57*, 3192–3196.

(41) Soupart, A.; Alary, F.; Heully, J. L.; Elliott, P. I.; Dixon, I. M. Theoretical Study of the Full Photosolvolysis Mechanism of [Ru(bpy)3]2+: Providing a General Mechanistic Roadmap for the Photochemistry of [Ru(NˆN)3]2+-Type Complexes toward Both Cis and Trans Photoproducts. *Inorg. Chem.* **2020**, *59*, 14679–14695.

(42) Lu, T. Concvar: A computer program for simulating concentration variation of complex chemical reactions. DOI: 10.26434/chemrxiv-2022-r6rh8-v2, 2022; `https://chemrxiv.org/engage/chemrxiv/article-details/61f6fdd9e4d9b811e2fd8773`.

(43) Stegelmann, C.; Andreasen, A.; Campbell, C. T. Degree of rate control: How much the energies of intermediates and transition states control rates. *J. Am. Chem. Soc.* **2009**, *131*, 13563.

(44) Campbell, C. T. The Degree of Rate Control: A Powerful Tool for Catalysis Research. *ACS Catal.* **2017**, *7*, 2770–2779.

(45) Huang, K.; Rhys, A. Theory of light absorption and non-radiative transitions in F-centres. *Proc. R. Soc. A* **1950**, *204*, 406–423.

(46) Peng, Q.; Shi, Q.; Niu, Y.; Yi, Y.; Sun, S.; Li, W.; Shuai, Z. Understanding the efficiency drooping of the deep blue organometallic phosphors: A computational study of radiative and non-radiative decay rates for triplets. *J. Mater. Chem. C* **2016**, *4*, 6829–6838.

(47) Caspar, J. V.; Meyer, T. J. Photochemistry of Ru(bpy)32+. Solvent Effects. *J. Am. Chem. Soc.* **1983**, *105*, 5583–5590.

(48) Kesharwani, M. K.; Brauer, B.; Martin, J. M. Frequency and zero-point vibrational energy scale factors for double-hybrid density functionals (and other selected methods): Can anharmonic force fields be avoided? *J. Phys. Chem. A* **2015**, *119*, 1701–1714.

(49) Nau, R. E.; Bösking, J.; Pannwitz, A. Compartmentalization Accelerates Photosensi-

tized NADH to NAD+ Conversion. *ChemPhotoChem* **2022**, *6*, e202200158.
